# The Role of Complement Activating Collectins and Associated Serine Proteases in Patients With Hematological Malignancies, Receiving High-Dose Chemotherapy, and Autologous Hematopoietic Stem Cell Transplantations (Auto-HSCT)

**DOI:** 10.3389/fimmu.2018.02153

**Published:** 2018-09-20

**Authors:** Anna S. Świerzko, Mateusz Michalski, Anna Sokołowska, Mateusz Nowicki, Łukasz Eppa, Agnieszka Szala-Poździej, Iwona Mitrus, Anna Szmigielska-Kapłon, Małgorzata Sobczyk-Kruszelnicka, Katarzyna Michalak, Aleksandra Gołos, Agnieszka Wierzbowska, Sebastian Giebel, Krzysztof Jamroziak, Marek L. Kowalski, Olga Brzezińska, Steffen Thiel, Jens C. Jensenius, Katarzyna Kasperkiewicz, Maciej Cedzyński

**Affiliations:** ^1^Laboratory of Immunobiology of Infections, Institute of Medical Biology, Polish Academy of Sciences, Łódź, Poland; ^2^Department of Hematology, Copernicus Memorial Hospital in Łódź Comprehensive Cancer Center and Traumatology, Łódź, Poland; ^3^Department of Bone Marrow Transplantation and Oncohematology, Cancer Center and Institute of Oncology, Gliwice, Poland; ^4^Department of Hematology, Medical University of Łódz, Łódź, Poland; ^5^Department of Hematology, Institute of Hematology and Transfusion Medicine, Warsaw, Poland; ^6^Department of Immunology and Allergy, Medical University of Łódz, Łódź, Poland; ^7^Department of Rheumatology, Medical University of Łódz, Łódź, Poland; ^8^Department of Biomedicine, Aarhus University, Aarhus, Denmark; ^9^Department of Microbiology, University of Silesia, Katowice, Poland

**Keywords:** CL-LK, collectin, complement, hematopoietic stem cell transplantation (HSCT), mannose-binding lectin (MBL), MASP, mutliple myeloma, lymphoma

## Abstract

We conducted a prospective study of 312 patients (194 with multiple myeloma, 118 with lymphomas) receiving high-dose conditioning chemotherapy and autologous hematopoietic stem cell transplantation (auto-HSCT). Polymorphisms of *MBL2* and *MASP2* genes were investigated and serial measurements of serum concentrations of mannose-binding lectin (MBL), CL-LK collectin and MASP-2 as well as activities of MBL-MASP-1 and MBL-MASP-2 complex were made. Serum samples were taken before conditioning chemotherapy, before HSCT and once weekly after (totally 4-5 samples); in minority of subjects also 1 and/or 3 months post transplantation. The results were compared with data from 267 healthy controls and analyzed in relation to clinical data to explore possible associations with cancer and with chemotherapy-induced medical complications. We found a higher frequency of MBL deficiency-associated genotypes (LXA/O or O/O) among multiple myeloma patients compared with controls. It was however not associated with hospital infections or post-HSCT recovery of leukocytes, but seemed to be associated with the most severe infections during follow-up. Paradoxically, high MBL serum levels were a risk factor for prolonged fever and some infections. The first possible association of *MBL2* gene 3′-untranslated region polymorphism with cancer (lymphoma) in Caucasians was noted. Heterozygosity for *MASP2* gene +359 A>G mutation was relatively frequent in lymphoma patients who experienced bacteremia during hospital stay. The median concentration of CL-LK was higher in myeloma patients compared with healthy subjects. Chemotherapy induced marked increases in serum MBL and MASP-2 concentrations, prolonged for several weeks and relatively slighter decline in CL-LK level within 1 week. Conflicting findings on the influence of MBL on infections following chemotherapy of myeloma and lymphoma have been reported. Here we found no evidence for an association between MBL deficiency and infection during the short period of neutropenia following conditioning treatment before HSCT. However, we noted a possible protective effect of MBL during follow-up, and suspected that to be fully effective when able to act in combination with phagocytic cells after their recovery.

## Introduction

Hematological cancers derive from various cells of the immune system. Multiple myeloma (MM), a plasma cell malignancy, is relatively common. It more often affects males than females and is usually diagnosed at age >55 years. Although incurable, treatment strategies including chemotherapy followed by autologous hematopoietic stem cell transplantation (auto-HSCT) allow marked prolongation of patients' life expectancy (1–5). The term “lymphoma” includes a variety of highly heterogeneous lymphoid malignancies. Hodgkin's lymphoma (HL) accounts for approximately 1/10 of all lymphomas at diagnosis. It is characterized by giant, multinucleated Reed-Sternberg and large mononuclear Hodgkin's cells (of B cell lineage). Incidence of HL peaks during early adulthood (20–34 years) and again at age >60 years; it is slightly commoner in males, and most newly diagnosed patients are successfully cured (6–9). Non-Hodgkin's lymphomas (NHL) are also more often diagnosed in men than in women (10–15). NHL usually arise from the B cells lineage (>40 subtypes; >80% of cases) although some T cell and NK cell lymphomas occur. Some NHL are histologically aggressive with a high mortality rate, but NHL are often curable when diagnosed and treated early.

Hematological malignancies obviously compromise the host immune response. Intensive chemotherapy and/or radiotherapy, adds further immunosuppression (mostly due to profound and prolonged neutropenia). High-dose chemotherapy and auto-HSCT is the standard first-line treatment for younger patients with multiple myeloma as well as standard second-line treatment in patients with Hodgkin's lymphoma and aggressive non-Hodgkin's lymphomas (1, 6, 10, 16).

Due to severe immunocompromise caused by both the disease and therapy, patients are at a high risk of morbidity and mortality due to infections. It is estimated that up to 80% of patients have fever, and more than one third suffer from well-defined infections (17–19). Septicemia is the commonest type of infection, and some patients are affected by pneumonia or alimentary tract infections (19). Post-HSCT reconstitution of bone marrow after conditioning therapy is usually attained in ~3 weeks, but full recovery of functional leukocytes takes longer (20, 21). During the period of aplasia, patients have to be isolated, carefully monitored and receive prophylactic/supportive medication (4, 18, 22).

Complement is a complex system of numerous components that constitutes a crucial branch of the immune response. It protects from a variety of infections and takes part in clearance of host cells undergoing apoptosis, necrosis or neoplastic transformation. However, dysregulation of its activation may result in chronic or systemic inflammation and possibly promotion of tumor growth. Therefore, involvement of complement may have opposing effects in cancer. Furthermore, some of its components may be considered as candidate diagnostic or prognostic biomarkers of the disease (23, 24).

Pattern recognition molecules known to activate complement *via* the lectin pathway are two collectins [mannose-binding lectin (MBL) and CL-LK, composed of two peptides: collectin-10 (CL-10, CL-L1) and collectin-11 (CL-11, CL-K1)] and ficolins (ficolin-1,-2,-3). They form complexes with MASP (MBL-associated serine proteases: MASP-1,-2,-3) and non-enzymatic related proteins (MAp19, MAp44) (25–27).

The aim of our investigation was to estimate possible association of complement-activating collectins (MBL, CL-LK) and associated serine proteases with cancer (MM, LYMPH) itself and with hospital infections after chemotherapy followed by auto-HSCT.

Single-nucleotide polymorphisms, localized to codons 52 (A/D), 54 (A/B), and 57 (A/C) of the first exon of the *MBL2* gene, influence both MBL serum level and its activity. The variant, dominant alleles (collectively designated O), are associated with lower MBL levels compared with the A (wild-type) one. Furthermore, promoter region polymorphisms (H/L and Y/X at positions−550 and−221, respectively) also determine MBL concentration to some extent, due to influence on gene expression. The O/O and LXA/O genotypes are considered to be associated with primary MBL deficiency [reviewed in (24)].

The role of MBL and clinical associations of its deficiency apparently depend on clinical context, patient's age, co-existence of other defects of the immune system, *etc*. Several reports and review papers suggested that *MBL2* A/O heterozygosity may be advantageous for the host. Relative high frequency of such genotypes in a variety of populations has been suspected to result from an evolutionary, selective trend (28–31). It was observed that *MBL2* O alleles (and especially O/O genotypes) are less common among centenarians than in general population. On the other hand, in this age group, a relatively low frequency of high MBL-producing haplotypes/genotypes was found. Similar trends were noted in octo- and nonagenarians (32, 33). Therefore, it was postulated that moderate MBL serum concentration may be favorable to healthy aging while both MBL deficiency and its hyper-reactivity may be deleterious for the host. The first was related to variety of infectious which, especially when severe or recurrent, may lead to the worsening of quality of life and therefore to shortening of life expectancy. On the other hand, high MBL may favor autoimmunity and/or promote tumorigenesis. The latter is often associated with cachexia and fatal outcome. That issue becomes even more complicated when some opposing effects are considered: MBL deficiency may be a risk factor for development of cancer (like childhood acute lymphoblastic leukemia) while high MBL may facilitate some intracellular pathogens to enter their target cells [reviewed in (24, 33)].

Originally, Peterslund et al. (34) found a strong relationship between MBL deficiency and serious infections in a relatively small group of patients suffering from hematological malignancies (*n* = 54, including 18 with MM and 13 with NHL). Many subsequent studies failed to reproduce the strength of this relationship although positive results were reported in some studies but not others. It is clear that MBL does not have a universal role in preventing infections following chemotherapy, but any influence or lack of it is still controversial. Further studies are therefore needed and should be carried out prospectively with substantial numbers of patients. We have investigated a large (*n* = 194) series of multiple myeloma patients and compared them with a more heterogeneous group of lymphoma patients (*n* = 118), as well as healthy controls (*n* = 267). In addition to MBL, we have also extended our investigations to include CL-LK and MASP-2 since those factors are also relevant to collectin-mediated complement activation.

## Materials and methods

### Patients and controls

Three hundred and twelve patients suffering from hematological malignancies, undergoing auto-HSCT were recruited. This group included 194 persons diagnosed with multiple myeloma (MM; 95 females and 99 males; mean age: 58.9 ± 8.9 years), 27 with Hodgkin's lymphoma (HL; 6 females and 21 males; mean age: 40.8 ± 13.7) and 91 with non-Hodgkin's lymphomas (NHL; 40 females, 51 males; mean age: 51.6 ± 11.9). For analyses, HL and NHL groups were combined to form LYMPH (*n* = 118; mean age: 49.3 ± 13.1). The NHL group included patients with diffuse large B-cell lymphoma (DLBCL, *n* = 31), mantle cell lymphoma (MCL, *n* = 18), follicular lymphoma (FL, *n* = 13), Waldenström's macroglobulinemia (WM, *n* = 1), non-follicular (diffuse) lymphoma, unspecified (NFL, *n* = 21), marginal-zone lymphoma (mucosa-associated lymphoid tissue lymphoma, MALTL, *n* = 1), anaplastic large cell lymphoma, kinase negative (ALCL ALK-, *n* = 1), anaplastic large cell lymphoma, kinase positive (ALCL ALK+, *n* = 1), angioimmunoblastic T*-*cell lymphoma (AITL, *n* = 2), peripheral T-cell lymphoma (PTCL, *n* = 1), hepatosplenic T-cell lymphoma (HSTL, *n* = 1). In total, the group of patients included 141 females and 171 males; mean age was 55.2 ± 11.7 years; age range: 21–73). Basic demographic data are summarized in Table 1.

**Table 1:** Basic characterisation of patients and controls.

	**Group**		
	**Multiple myeloma (MM)**	**Lymphoma (LYMPH)**	**Control (C)**
N	194	118	267
Sex (F/M)	95/99	46/72	174/93
Age (years)			
mean ± SD range	58.9 ± 8.9 25-71	49.3 ± 13.1 21–73	48 ± 13 18–84
Conditioning therapy (n)	MEL-200 (156) MEL-140 (19) MEL-100 (7) Radiotherapy alone (12)	BEAM (88) BeAM (12) Bendamustine (4) CTX (13) TBC (1)	Not applicable
Complications (n) Infections with bacteremia with no bacteremia Febrile neutropenia None of above	74 45 30 39 81	72 35 38 10 36	Not applicable

With the exception of 12 persons suffering from MM undergoing radiotherapy alone, patients were treated with conditioning high-dose chemotherapy before transplantation (Table 1). In 28 patients (24 LYMPH and 4 MM), both chemotherapy and radiotherapy were used. Standard treatment for multiple myeloma was MEL-200 (melphalan at dosage 200 mg/m^2^) although in some instances lower doses (MEL-140, MEL-100) were administered. The majority of LYMPH patients were treated with BEAM: carmustine (300 mg/m^2^), etoposide (800 mg/m^2^), cytarabine (1600 mg/m^2^), melphalan (140 mg/m^2^), accompanied, or not by radiotherapy. For the remaining, BeAM (carmustine replaced with bendamustine), bendamustine (alone or accompanied by radiation), cyclophosphamide (CTX) and radiotherapy or TBC combination (thiotepa, busulfan, cyclophosphamide) were used (Table 1).

During hospital stay, crucial clinical parameters like white blood cell (WBC) count, absolute neutrophil count (ANC), platelet (PLT) count, inflammatory markers [C-reactive protein (CRP), fibrinogen (FBG), and procalcitonin (PCT)] levels and incidence of complications [infections (associated with bacteremia or not), febrile neutropenia (FN); duration of fever >38°C] were recorded and used for analyses. Etiological agents of infections are listed in Supplementary Table 1.

One hundred age-matched controls (unrelated volunteers with no history of cancer, autoimmune diseases, or recurrence of infections) were recruited. Furthermore, DNA/serum samples from 167 healthy adult subjects collected for our previous projects were used, therefore control group included 267 individuals (174 females and 93 males; mean age: 48 ± 13; age range: 18–84). Basic data are presented in Table 1. The study was approved by the Ethics Committee of the Medical University of Lodz. The written informed consent from patients/controls was obtained. This work conforms to the provisions of the Declaration of Helsinki.

### Blood and serum samples

Blood samples for DNA extraction were taken into citrated tubes before chemotherapy and stored at −80°C. DNA was extracted with the use of GeneMATRIX Quick Blood Purification Kit (EURx Ltd, Gdansk, Poland), according to the manufacturer's protocol. Samples for serum isolation were taken into tubes with clot activator before chemotherapy (sample 1), before HSCT (sample 2; usually 3–7 days later) and once weekly till hospital discharge (sample 3: usually 7 days after HSCT; mean 7.0 ± 0.8; sample 4: usually 14 days after HSCT; mean 13.6 ± 1.2; in some patients sample 5: 21 days after HSCT; mean 20.3 ± 2). Sixty-three patients were additionally sampled~45 days (mean 45.3 ± 7.6; sample 45) while 59 were additionally sampled at ~100 days (mean 104.1 ± 19.3; sample 100) after HSCT, during control examination. Among them, 40 persons were sampled at “45” and “100” points. Sera were stored at −80°C until testing.

### *MBL2* genotyping

Promoter (H/L, at position−550, rs11003125 and Y/X, at position−221, rs7096206) and exon 1 (A/D, codon 52, rs5030737; A/B, codon 54, rs1800450, and A/C, codon 57, rs1800451) single nucleotide polymorphisms of the *MBL2* gene were investigated as previously described (35). SNPs of the same gene, located within the 3′-untranslated region of the exon 4: Ex4-1483 T>C (rs10082466), Ex4-1260 C>T (rs56009657), Ex4-1067 G>A (rs10824792), Ex4-1064 G>T (rs35327474), Ex4-1063 G>T (rs35768126), Ex4-1047 T>G (rs12254577), Ex4-939 T>C (rs774307463), Ex4-901 A>G (rs2120132), Ex4-879 A>C (rs2120131), Ex4-845 C>T (rs2165813), Ex4-718 G>T (rs2099903) and Ex4-710 A>G (rs2099902) were investigated by direct sequencing. Briefly, PCRs were run on a C1000 Thermal Cycler (Bio-Rad, Hercules, CA, USA), using appropriate spanning primers (designed with the use of PRIMER3 software, http://bioinfo.ut.ee/primer3/):

forward: 5′-TTTCCCCATGGTTTTAATCTG-3′,

reverse: 5′-TCACTAAAACCACCAAAACAAGA-3′,

under the following conditions: 96°C for 5 min, then 35 cycles (96°C for 40 s, 60°C for 30 s, 72°C for 45 s) and finally 72°C for 5 min. The PCR products were purified with the help of EPPiC - Enzymatic Post-PCR Immediate Cleanup (A&A Biotechnology, Gdynia, Poland). Samples thus prepared were directly used as templates for sequencing, performed using the GeneAnalizer-3000 sequencer (Applied Biosystems, Foster City, CA, USA), reverse primer, BrightDyeTerminator Cycle Sequencing kit (NimaGen BV, Nijmegen, The Netherlands) and BDX64 Sequencing Enhancement Buffer (MCLab, San Francisco, CA, USA) according to the manufacturer's instructions. Sequence electropherograms were visually inspected to confirm base calling at the SNP sites using novoSNP (36).

### *MASP2* genotyping

The presence of missense mutation +359 A>G (Asp120Gly) located within exon 3 of the *MASP2* gene, rs72550870) was investigated with the use of PCR-RFLP method, as described previously (37).

For an investigation of +1111 A>C (exon 10, Asp371Tyr, rs12711521) polymorphism, the PCR-RFLP procedure was employed. PCR reactions were run on a C1000 Thermal Cycler (Bio-Rad), using primers designed with the use of PRIMER3 software:

forward: 5′-TTATTTTCAGACCATGGGGG-3′,

reverse: 5′-TCTGGGTGGTTTCAAATTCC-3′.

The following conditions were applied: 95°C for 3 min, then 35 cycles (95°C for 30 s, 61°C for 20 s and 72°C for 30 s), followed by a final elongation (72°C for 5 min). After that, the PCR products were treated with MboI enzyme (Fermentas), (37°C, 2 h). The digestion products were further analyzed on a 6% polyacrylamide gel. The PCR product (522 bp) corresponding to C variant has two digestion sites while that corresponding to A allele has only one. Consequently, the first underwent cleavage into three (286, 176, and 60 bp) while the second was cleaved into two (286 and 237 bp) fragments.

### MBL quantification and determination of MBL-MASP activity

MBL concentration/activity was measured by ELISA based on binding to solid-phase mannan and detection using monoclonal anti-human MBL antibody (HYB131-1, BioPorto Diagnostics, Copenhagen, Denmark) as previously described (38). MBL-MASP-1 complex activity (39, 40) and MBL-MASP-2 activity (40, 41), were measured using a fluorescence method and an ELISA, respectively. VPR-AMC peptide (Bachem, Bubendorf, Switzerland) was used as the substrate for MASP-1 (39). Rabbit polyclonal anti-human C4c Abs (Dako, Glostrup, Denmark) and corresponding goat HRP-conjugated secondary antibodies (Dako) were used for detection of deposited C4 products after incubation of the serum sample on mannan-coated wells, for an estimation of MBL-MASP-2 complex activity (40). MBL deficiency was taken as 150 ng/ml (42, 43). “High” MBL concentration (>3000 ng/ml) was chosen based on the value corresponding to the 95th percentile determined for the control group (2978 ng/ml). For MBL-MASP-1 or-2 complex activities, the value of 1 U/ml was arbitrarily assigned for the standard serum (38, 40).

### Assay for CL-LK

CL-LK concentration was determined by sandwich TRIFMA, as described (44). Mouse anti-human CL-10 monoclonal antibody (clone 4F97D) was used for coating while biotinylated rabbit anti-human CL-10 mAb (clone P1), followed by Eu^3+^-labeled streptavidin (Perkin Elmer, Waltham, MA, USA) was used for detection. “Low” and “high” CL-LK levels were arbitrarily defined as < 242 ng/ml (corresponding to the 10th percentile within control group) and >583 ng/ml (95th percentile determined for controls), respectively.

### Assay for MASP-2

MASP-2 levels were determined by TRIFMA, as described by Moller-Kristensen et al. (45), with slight modification (37). Rat antibodies of clone 8B were used for coating, while those of clone 6G12 (biotinylated) were used for detection. “Low” and “high” MASP-2 levels (based on 10 and 95th percentiles within C group) were arbitrarily defined as < 172 ng/ml and >627 ng/ml, respectively.

### Statistical analysis

The Statistica (version 13.1, Dell Poland) software package was used for data management and statistical calculations. The medians of protein concentrations were compared using the Mann-Whitney *U*-test. It was chosen because MBL, MASP-2, MBL-MASP-1, and MBL-MASP-2 values were not normally distributed (not shown). The same test was employed for CL-LK (normally distributed), to make the analyses comparable. Changes during hospital stay and after discharge were analyzed by Friedman's ANOVA test. The frequencies of low or high levels, as well as genotypes/alleles were compared by two-sided Fischer's exact test (or χ^2^ when appropriate). Correlations were determined by Spearman's test. *P* < 0.05 were considered statistically significant.

## Results

### *MBL2* gene polymorphisms located within promoter region and exon 1

The frequency of A/A homozygotes was similar in MM, LYMPH and C groups. However, MBL deficiency-associated genotypes (LXA/O, O/O) were significantly more common in patients with multiple myeloma (Table 2). Those genotypes did not appear in the short term (2–3 weeks after HSCT) to be associated with higher susceptibility to infection as their frequency did not differ significantly between patients experiencing hospital infections/febrile neutropenia compared with patients free of those complications [MM: 15/114 (13.2%) vs. 18/80 (22.5%, *p* = 0.12); LYMPH: 10/82 (12.2%) vs. 4/36 (11.1%, *p* = 1)]. The high MBL-conferring genotypes (YA/YA) were associated with a trend toward more prolonged fever during the neutropenic period, with 47.1% of patients having fever of more than 4 days duration being YA/YA, compared with 24.1% having fever for less than 4 days and 30.1% who had no infection or FN. The LYMPH group differed from the MM patients in having a high frequency of the uncommon C allele (codon 57 SNP) (0.025 vs. 0.007 for C; *p* = 0.049), but this was not associated with hospital infections.

**Table 2:** Frequency of *MBL2* genotypes (SNP located within promoter and exon 1) within major groups.

**Genotype**	**Group**		
	**C**	**MM**	**LYMPH**
A/A	180 (69.5)	132 (68)	80 (67.8)
YA/O	53 (20.5)	29 (14.9)	24 (20.3)
XA/O or O/O	26 (10)	33 (17)^*^	14 (11.9)

*Percentages are shown in parentheses*.

* *p = 0.034; OR 1.84; 95%CI (1.06-3.19) (vs. C)*.

One hundred and two MM patients were followed-up for at least 6 months, of which six were hospitalized due to severe infections. One was O/O and 2 were LXA/O; two of them died. Other 3 persons (1 non-survivor) had A/A genotypes. Similarly, from 59 LYMPH patients that were followed-up, three had severe infections: two were MBL-deficient (O/O; one non-survivor) and one A/A (survivor).

### *MBL2* gene polymorphisms located within 3′-utr region (exon 4)

Twelve polymorphic sites located within 3′-UTR region of the *MBL2* gene were analyzed. That allowed identification of 27 various genotypes, of which the 7 most common accounted for nearly 95% of controls and cases (Table 3). The genotype 1 (gt1) was commonest in the C and MM groups, but for the LYMPH group, genotype 2 was the most frequent. The difference between LYMPH and C groups with regard to genotype 1 (23.7 vs. 37.2%; *p* = 0.01) was statistically significant, possibly the first association of a *MBL2* gene 3′-untranslated region polymorphism with cancer to be observed in Caucasians.

**Table 3:** Frequency of *MBL2* genotypes (SNP located within exon 4 3′-UTR) within major groups.

**Genotype**	**Polymorphism**												**Group**		
	**Ex4-710**	**Ex4-718**	**Ex4-845**	**Ex4-879**	**Ex4-901**	**Ex4-939**	**Ex4-1047**	**Ex4-1063**	**Ex4-1064**	**Ex4-1067**	**Ex4-1260**	**Ex4-1483**	**C (*n* = 255)**	**MM (*n* = 194)**	**LYMPH (*n* = 118)**
gt1	A/A	G/G	C/C	A/A	A/A	T/T	T/T	G/G	G/G	A/A	C/C	T/T	95 (37.2)	59 (30.4)	28 (23.7)^*^
gt2	A/G	G/T	C/T	A/C	A/G	T/T	T/T	G/G	G/G	A/G	C/C	T/C	66 (25.9)	44 (22.7)	40 (33.9)
gt3	A/A	G/G	C/C	A/A	A/A	T/T	T/T	G/G	G/G	A/G	C/C	T/T	47 (18.4)	40 (20.6)	29 (24.6)
gt4	A/G	G/T	C/T	A/C	A/G	T/T	T/T	G/G	G/G	G/G	C/C	T/C	15 (5.9)	17 (8.8)	6 (5.1)
gt5	A/A	G/G	C/C	A/A	A/A	T/T	T/T	G/G	G/G	G/G	C/C	T/T	9 (3.5)	8 (4.1)	4 (3.4)
gt6	G/G	T/T	T/T	C/C	G/G	T/T	T/T	G/G	G/G	G/G	C/C	C/C	8 (3.1)	9 (4.6)	3 (2.5)
gt7	A/A	G/G	C/C	A/A	A/A	T/C	T/T	G/G	G/G	A/A	C/C	T/T	4 (1.6)	2 (1)	3 (2.5)
gt8	A/A	G/G	C/C	A/A	A/A	T/T	T/T	G/G	G/G	G/G	C/T	T/T	2 (0.8)	0	0
gt9	A/G	G/T	C/T	A/C	A/G	T/T	T/T	G/G	G/G	G/G	C/C	T/T	1 (0.4)	2 (1)	0
gt10	A/A	G/G	C/C	A/A	A/A	T/T	T/T	G/T	G/T	G/G	C/C	T/T	1 (0.4)	1 (0.5)	0
gt11	A/A	G/G	C/C	A/A	A/A	T/T	T/T	G/T	G/T	A/G	C/C	T/T	1 (0.4)	0	1 (0.8)
gt12	A/A	G/G	C/T	A/C	A/G	T/T	T/T	G/G	G/G	A/G	C/C	T/T	1 (0.4)	0	0
gt13	A/G	G/T	C/T	A/C	A/G	T/T	T/T	G/G	G/G	A/G	C/C	T/T	1 (0.4)	1 (0.5)	2 (1.7)
gt14	A/A	G/G	C/C	A/A	A/A	T/T	T/T	G/G	G/G	G/G	C/C	T/C	1 (0.4)	0	0
gt15	A/G	G/G	C/C	A/A	A/G	T/T	T/T	G/G	G/G	A/G	C/C	T/C	1 (0.4)	0	0
gt16	G/G	T/T	T/T	C/C	G/G	T/T	T/T	G/G	G/G	G/G	C/C	T/C	1 (0.4)	1 (0.5)	0
gt17	A/G	G/T	C/T	A/C	A/G	T/C	T/T	G/G	G/G	A/G	C/C	T/C	1 (0.4)	1 (0.5)	1 (0.8)
gt18	A/G	G/T	C/T	A/C	A/G	T/T	T/T	G/G	G/G	A/A	C/C	T/T	0	1 (0.5)	0
gt19	A/A	G/G	C/C	A/A	A/A	T/C	T/T	G/G	G/G	A/G	C/C	T/T	0	1 (0.5)	0
gt20	A/G	G/T	C/T	A/C	A/G	T/T	T/T	G/T	G/T	G/G	C/C	T/C	0	1 (0.5)	0
gt21	A/G	G/G	C/T	A/C	A/G	T/T	T/T	G/G	G/G	A/G	C/C	T/C	0	1 (0.5)	0
gt22	G/G	G/G	T/T	C/C	G/G	T/T	T/T	G/G	G/G	G/G	C/C	C/C	0	1 (0.5)	0
gt23	A/A	G/G	C/C	A/A	G/G	T/T	T/T	G/T	G/T	A/G	C/C	T/T	0	1 (0.5)	0
gt24	A/A	G/G	C/C	A/A	A/A	T/T	T/G	A/A	A/A	C/C	C/C	T/T	0	1 (0.5)	0
gt25	A/G	G/T	C/T	A/C	A/G	T/T	T/T	A/G	A/C	C/T	C/T	T/C	0	1 (0.5)	0
gt26	A/G	G/T	C/T	A/C	A/G	T/T	T/T	A/G	A/C	C/T	C/C	C/C	0	1 (0.5)	0
gt27	A/A	G/G	C/C	A/A	A/A	T/T	T/T	A/A	A/A	C/C	C/T	T/T	0	0	1 (0.8)

*Percentages are shown in parentheses. Differences in comparison with gt1 are marked in red*.

* *p = 0.01; OR 0.52; 95%CI (0.32-0.86) (vs. C)*.

### *MASP2* gene polymorphisms

When the +359 A>G mutation was investigated, no variant homozygote (and therefore MASP-2/MAp19 deficient) was found among individuals recruited to the study. The number of heterozygotes (Table 4) did not differ among the major (C, MM, LYMPH) groups, although a trend toward higher incidence of heterozygosity was noted within LYMPH group (Table 4). That primarily reflected relatively high frequency of A/G genotype among LYMPH patients who had bacteremia during hospital stay [8/34 (23.5%); *p* < 0.04 vs. C group].

**Table 4:** Frequency of *MASP2* genotypes (corresponding to polymorphisms +359 A>G and +1111 A>C SNP) within major groups.

**Group**	**Polymorphism**	
	**+359 A/G**	**+1111 A>C**
C	A/A: 233 (90)	A/A: 179 (69.6)
	A/G: 26 (10)	A/C: 69 (26.8)
	G/G: –	C/C: 9 (3.5)
MM	A/A: 175 (90.2)	A/A: 125 (64.4)
	A/G: 19 (9.8)	A/C: 65 (33.5)
	G/G: –	C/C: 4 (2.1)
LYMPH	A/A: 101 (85.6)	A/A: 91 (77.1)
	A/G: 17 (14.4)	A/C: 26 (22)^*^
	G/G: –	C/C: 1 (0.8)^*^

*Percentages are shown in parentheses*.

* *Number of C allele carriers (A/C or C/C genotype) significantly lower in comparison with MM patients: p = 0.023; OR 1.86; 95% CI (1.11-3.13)*.

For another *MASP2* SNP, +1111 A>C, no differences between patients and controls were found. However, this variant allele was commoner within the LYMPH patients than within the MM group (*p* = 0.023) (Table 4).

### Serum MBL concentration and activity of its complexes with serine proteases

**Figure 1:**
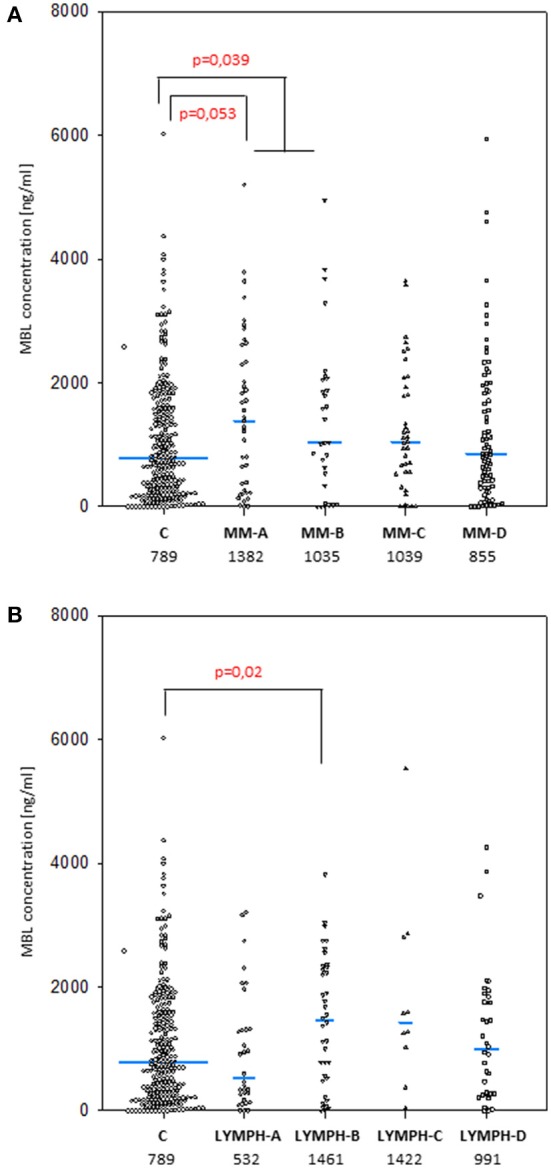
Mannose-binding lectin serum concentrations in patients (before conditioning chemotherapy) and controls. Blue bars present median values (given below group descriptions). **(A)** controls (C) vs. multiple myeloma (MM) patients; **(B)** controls vs. lymphoma (LYMPH) patients. MM-A, LYMPH-A: patients who experienced infections with proven bacteremia; MM-B, LYMPH-B: patients who experienced infections with no bacteremia; MM-C, LYMPH-C: patients who experienced febrile neutropenia; MM-D, LYMPH-D: patients who experienced none of afore-mentioned complications during hospital stay.

The median values determined for samples taken before chemotherapy did not differ significantly in either MM (1027 ng/ml; *n* = 187) or LYMPH (1072 ng/ml; *n* = 116) groups when compared with that found for healthy controls (789 ng/ml; *n* = 265) (Figure 1). However, the median was higher in MM patients who later experienced hospital infections (associated or not with bacteremia) and LYMPH patients who suffered from infections without bacteremia (Figure 1A).

As was expected from numerous other studies, the median serum MBL for males and females in the healthy controls were virtually identical (789 vs. 787 ng/ml), and the higher values found in the LYMPH group also showed no sex difference (1090 vs. 1054 ng/ml). However, male subjects in the MM group had significantly higher (1213 vs. 729 ng/ml; *p* = 0.002) values, and consequently male MM patients had higher average serum MBL than male controls (*p* = 0.032).

**Figure 2:**
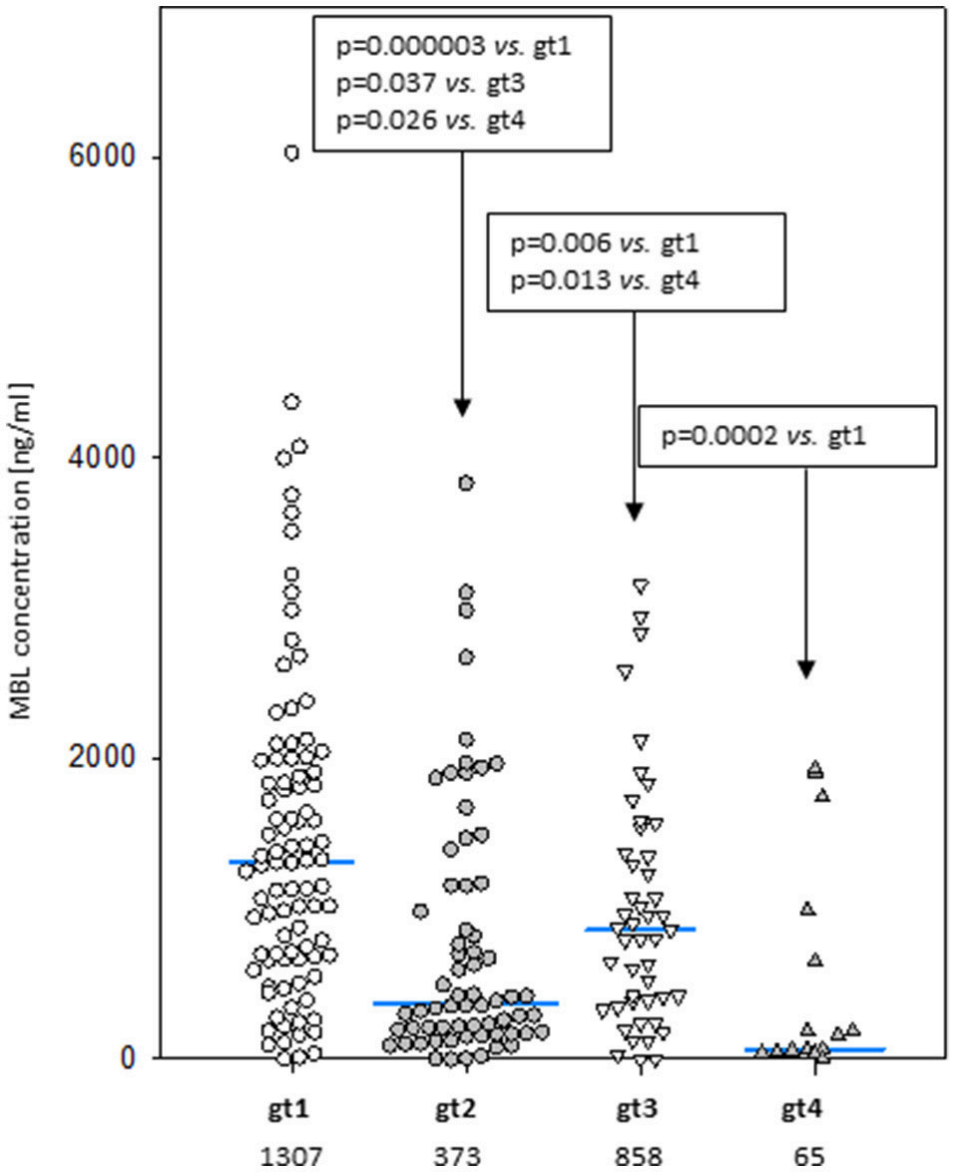
Mannose-binding lectin serum concentrations in healthy controls carrying most common 3′-UTR *MBL2* genotypes (gt1-gt4; details are given in Table 3). Blue bars present median values (given below group descriptions).

Despite well-known association between *MBL2* SNP located within promoter and exon 1 with MBL concentration (not shown), an influence of exon 4 polymorphisms has been observed. When the four most common genotypes were compared within the control group, significant differences in corresponding median MBL levels were found (Figure 2). Comparison of patients and controls showed MBL concentrations significantly higher in patients with genotype 3 for both MM and LYMPH groups (Table 5).

**Table 5:** Serum MBL concentrations in controls and patients (sample 1), corresponding to most common *MBL2* genotypes (basing on 3′-UTR polymorphisms).

**Genotype**	**Group (n)**	**MBL concentration (ng/ml)**		**Statistical significance (vs. C)**
		**Median**	**IQR**	
gt1	C (93)	1307	683–1969	–
	MM (59)	1681	804–2673	*p* = 0.066
	LYMPH (27)	1745	1039–2280	*p* = 0.12
gt2	C (66)	373	181–1154	-
	MM (41)	426	233–1213	*p* = 0.53
	LYMPH (40)	466	188–1479	*p* = 0.67
gt3	C (47)	858	384–1360	-
	MM (39)	1199	976–1858	*p* = 0.016
	LYMPH (28)	1285	880–1875	*p* = 0.016

**Figure 3:**
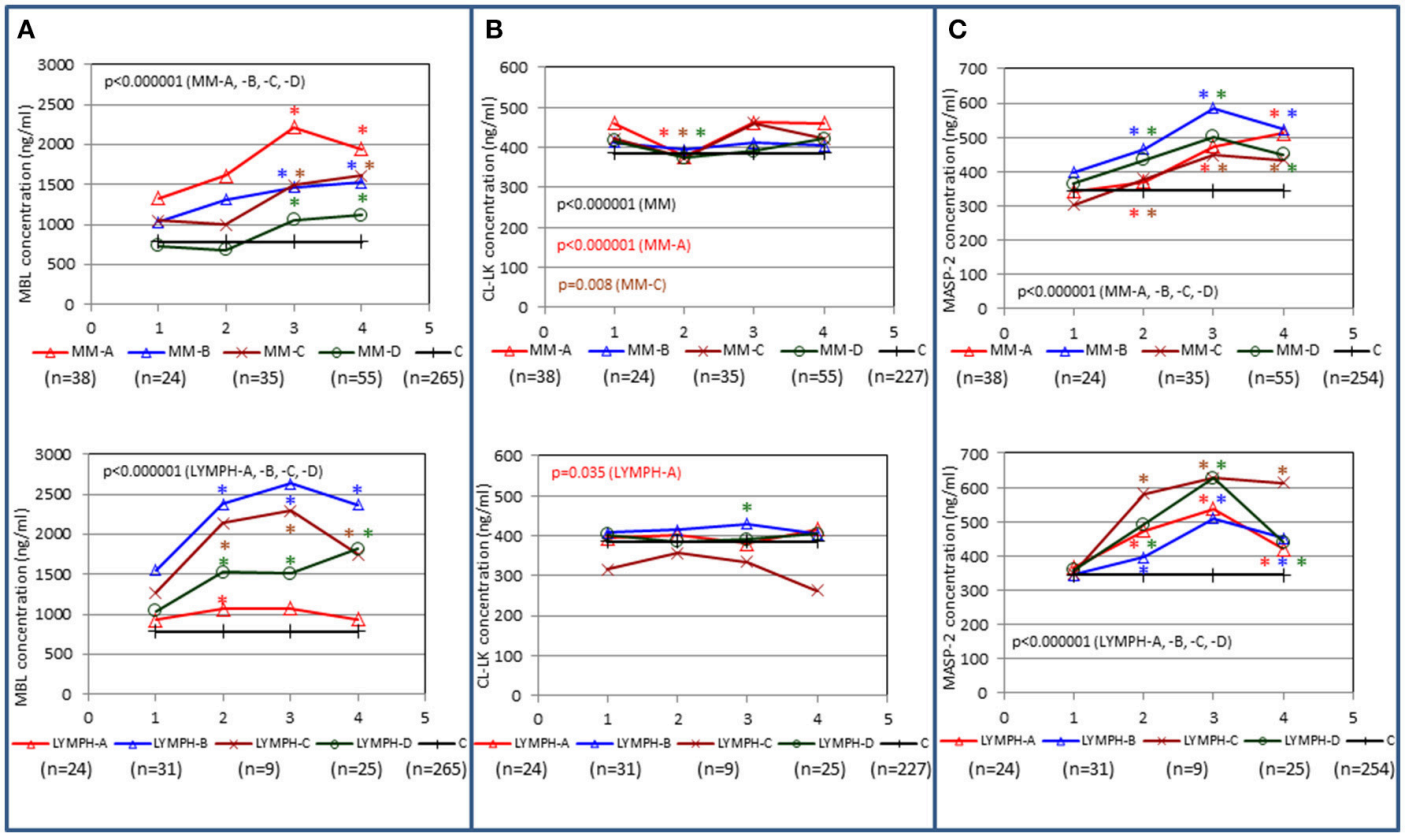
Changes in concentrations of MBL **(A)**, CL-LK **(B)**, and MASP-2 **(C)** in patients, during treatment. 1–blood taken directly before conditioning chemotherapy; 2 – blood taken directly before autologous hematopoietic stem cell transplantation; 3–blood taken 1 week after HSCT; 4—blood taken 2 weeks after HSCT. Data from sample 5 (3 weeks after HSCT) are not shown due to relatively low number of cases within each subgroup. Median values for each time-point are presented. MM-A, LYMPH-A: patients who experienced infections with proven bacteremia; MM-B, LYMPH-B: patients who experienced infections with no bacteremia; MM-C, LYMPH-C: patients who experienced febrile neutropenia; MM-D, LYMPH-D: patients who experienced none of afore-mentioned complications during hospital stay. C—controls (sampled once). Statistics: given *p*-values regard to Friedman's ANOVA while asterisks (in colors corresponding to curves) mark significant differences in comparison with sample 1 (Wilcoxon's paired sample test). Graphs show data from complete sets (MBL levels measured in all 1-4 samples) only. Numbers of patients are given in parentheses.

**Figure 4:**
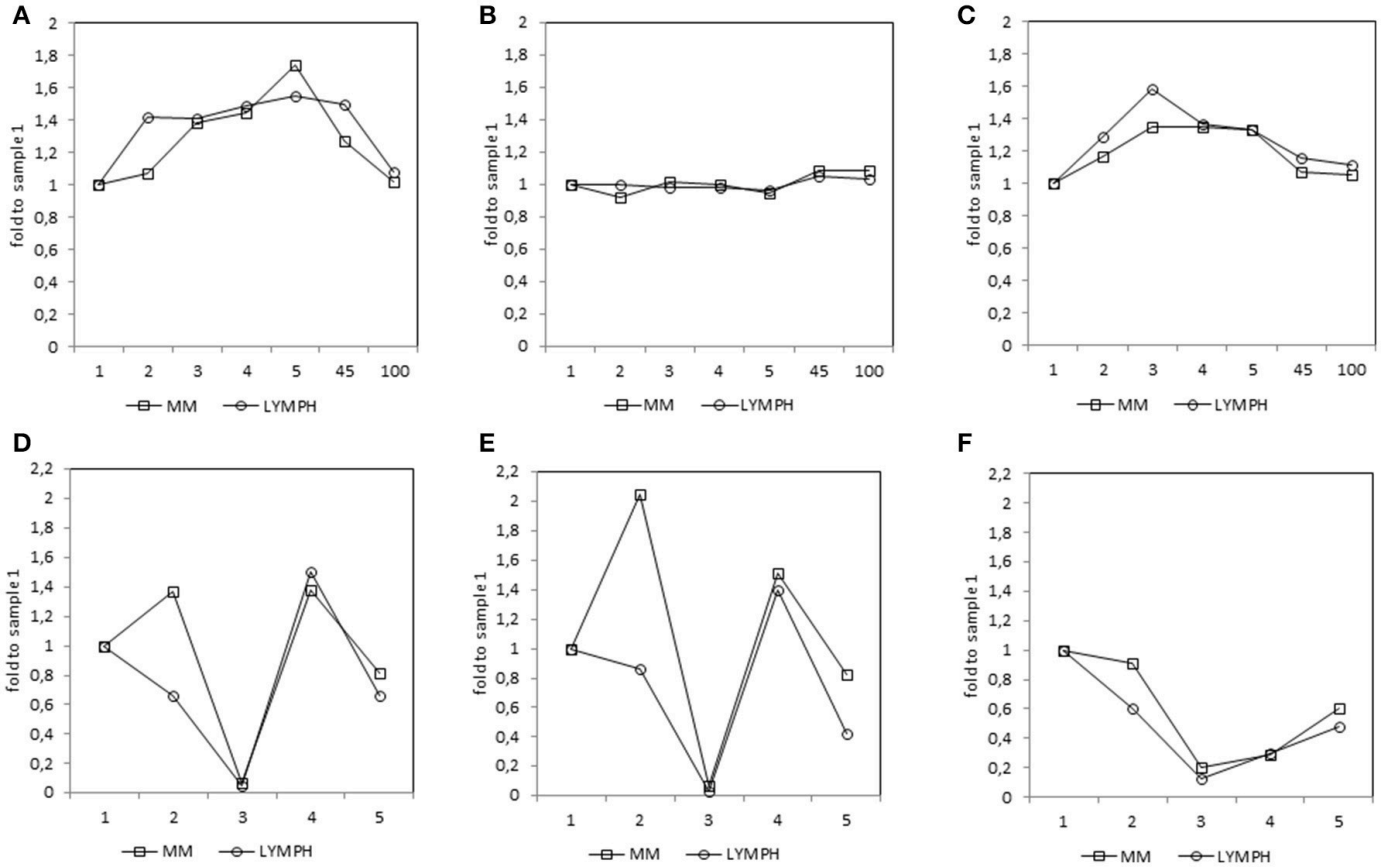
Relative (median fold to sample 1) changes in concentrations of MBL **(A)**, CL-LK **(B)**, and MASP-2 **(C)** and blood morphology: white blood cells (WBC) count **(D)**, absolute neutrophil count (ANC) **(E)** and platelet (PLT) count **(F)** in patients, during treatment. 1—blood taken directly before conditioning chemotherapy; 2—blood taken directly before autologous hematopoietic stem cell transplantation; 3—blood taken 1 week (mean 7.0 ± 0.8 days) after HSCT; 4—blood taken 2 weeks after HSCT (mean 13.6 ± 1.2 days); 5—blood taken 3 weeks after HSCT (mean 20.3 ± 2 days); 45—blood taken 45 days after HSCT (mean 45.3 ± 7.6 days); 100—blood taken 100 days after HSCT (mean 104.1 ± 19.3 days).

In both MM and LYMPH groups, MBL concentrations underwent significant changes (*p* < 0.000001; Friedman's ANOVA test) in relation to treatment (Figure 3A). The changes were generally more evident in persons experiencing complications during hospital stay (with the exception of LYMPH patients with bacteremias). (Figure 3A). Data from samples taken during control examinations (although from limited number of patients) demonstrated that the concentration of this lectin ~100 days post-HSCT practically returns to the initial (sample 1) value (Figure 4A). In the case of patients suffering from lymphomas, MBL increased more rapidly than in those with diagnosed MM, probably depending on chemotherapy used (BEAM vs. MEL) (Figure 3A). Again, a decrease was observed after discharge and data from sample 100 were comparable to those from sample 1 (Figure 4A).

Unexpectedly, very high MBL concentration (>3000 ng/ml) before treatment seemed to be associated with higher risk of hospital-acquired infection in MM patients. Such a high level of the protein was found in 9 of 71 (12.7%) MM patients who experienced infections, compared with 13 of 265 controls (4.9%; OR 2.81; *p* = 0.029). No such relationship was found for the LYMPH group (8/116 cases, 6.9%). No difference was found in the frequency of serum MBL deficiency (<150 ng/ml) between controls (17.7%) and patients (MM: 17.6%; LYMPH: 16.4%). independently of complications (not shown).

As expected, since correlation coefficients between serum MBL and activity of its complexes with MASP-1 or MASP-2 were ≥0.9, the latter activities gave similar results (Supplementary Figure 1). Moreover, observed MBL variations in MM patients, slightly but significantly, inversely correlated with variations in WBC, ANC, PLT counts (changes in leukocyte counts are demonstrated in Figures 4D–F). In contrast, relatively strong, positive correlations with concentrations of such markers of inflammation as CRP or PCT (but not FBG) were found (Table 6). It should be mentioned that WBC/ANC/PLT were checked routinely and regularly (taking samples 1-5 was coordinated with that) while concentrations of inflammatory proteins (especially FBG) were tested occasionally, in the case of morbid indications. Therefore, even relatively high correlation coefficient not always was associated with statistical significance (Table 6).

**Table 6:** Correlations (Spearman's) between variations (samples 1–5) of concentrations/activities of tested proteins/complexes and selected clinical parameters (significant positive and inverse correlations marked with blue and red, respectively).

**Tested factor**	**Blood cell counts**			**Markers of inflammation**		
	**WBC**	**ANC**	**PLT**	**CRP**	**FBG**	**PCT**
**A**						
MBL	*r* = −0.09	*r* = −0.11	*r* = −0.08	*r* = 0.09	*r* = 0.07	*r* = 0.12
	*p* = 0.013	*p* = 0.002	*p* = 0.029	*p* = 0.043	*p* = 0.63	*p* = 0.63
	*n* = 792	*n* = 784	*n* = 790	*n* = 518	*n* = 49	*n* = 20
MBL-MASP-1	*r* = −0.09	*r* = −0.11	*r* = −0.07	*r* = 0.08	*r* = 0.06	*r* = 0.14
	*p* = 0.015	*p* = 0.002	*p* = 0.052	*p* = 0.09	*p* = 0.69	*p* = 0.56
	*n* = 791	*n* = 783	*n* = 789	*n* = 517	*n* = 49	*n* = 20
MBL-MASP-2	*r* = −0.06	*r* = −0.09	*r* = −0.07	*r* = 0.1	*r* = 0.11	*r* = 0.12
	*p* = 0.11	*p* = 0.019	*p* = 0.072	*p* = 0.036	*p* = 0.46	*p* = 0.61
	*n* = 744	*n* = 736	*n* = 743	*n* = 487	*n* = 48	*n* = 20
CL-LK	*r* = −0.08	*r* = −0.09	*r* = −0.04	*r* = 0.1	*r* = 0.05	*r* = −0.13
	*p* = 0.022	*p* = 0.01	*p* = 0.21	*p* = 0.025	*p* = 0.76	*p* = 0.57
	*n* = 792	*n* = 784	*n* = 790	*n* = 518	*n* = 49	*n* = 20
MASP-2	*r* = −0.1	*r* = −0.09	*r* = −0.2	*r* = 0.2	*r* = −0.003	*r* = −0.08
	*p* = 0.004	*p* = 0.015	*p* < 0.000001	*p* = 0.000005	*p* = 0.98	*p* = 0.75
	*n* = 790	*n* = 782	*n* = 788	*n* = 516	*n* = 49	*n* = 20
**B**						
MBL	*r* = −0.03	*r* = −0.03	*r* = −0.09	*r* = 0.12	*r* = 0.03	*r* = 0.35
	*p* = 0.51	*p* = 0.53	*p* = 0.06	*p* = 0.044	*p* = 0.82	*p* = 0.015
	*n* = 463	*n* = 443	*n* = 462	*n* = 302	*n* = 72	*n* = 48
MBL-MASP-1	*r* = −0.02	*r* = 0.002	*r* = −0.07	*r* = 0.05	*r* = 0.06	*r* = 0.35
	*p* = 0.62	*p* = 0.97	*p* = 0.12	*p* = 0.41	*p* = 0.65	*p* = 0.014
	*n* = 462	*n* = 442	*n* = 461	*n* = 301	*n* = 72	*n* = 48
MBL-MASP-2	*r* = −0.005	*r* = 0.01	*r* = −0.08	*r* = 0.1	*r* = 0.15	*r* = 0.42
	*p* = 0.92	*p* = 0.84	*p* = 0.01	*p* = 0.071	*P* = 0.21	*p* = 0.003
	*n* = 441	*n* = 421	*n* = 440	*n* = 291	*n* = 71	*n* = 48
CL-LK	*r* = −0.02	*r* = 0.01	*r* = 0.06	*r* = 0.03	*r* = 0.05	*r* = 0.33
	*p* = 0.63	*p* = 0.77	*p* = 0.19	*p* = 0.56	*p* = 0.65	*p* = 0.02
	*n* = 463	*n* = 443	*n* = 462	*n* = 302	*n* = 72	*n* = 48
MASP-2	*r* = −0.27	*r* = −0.27	*r* = −0.31	*r* = 0.32	*r* = 0.13	*r* = 0.27
	*p* < 0.000001	*p* < 0.000001	*p* < 0.000001	*p* < 0.000001	*p* = 0.28	*p* = 0.066
	*n* = 463	*n* = 443	*n* = 462	*n* = 302	*n* = 72	*n* = 48

*A – patients suffering from multiple myeloma; B – patients suffering from lymphomas*.

### Concentration of CL-LK

**Figure 5:**
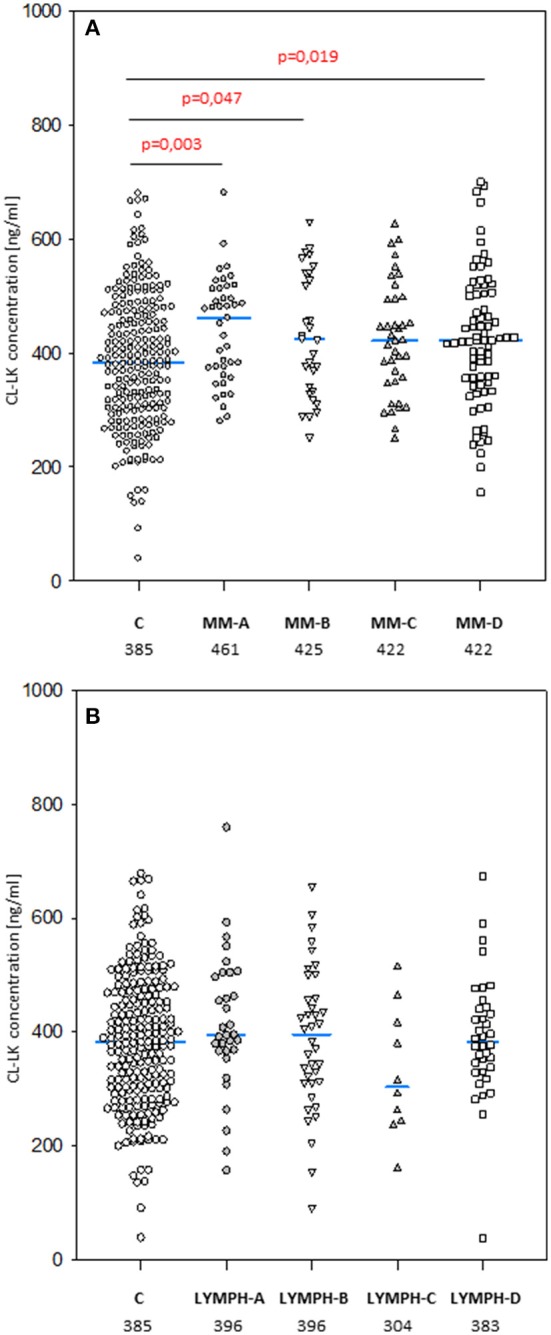
Collectin CL-LK serum concentrations in patients (before conditioning chemotherapy) and controls. Blue bars present median values (given below group descriptions). **(A)** controls (C) vs. multiple myeloma (MM) patients; **(B)** controls vs. lymphoma (LYMPH) patients. MM-A, LYMPH-A: patients who experienced infections with proven bacteremia; MM-B, LYMPH-B: patients who experienced infections with no bacteremia; MM-C, LYMPH-C: patients who experienced febrile neutropenia; MM-A, LYMPH-A: patients who experienced none of afore-mentioned complications during hospital stay.

The median level of CL-LK in pre-treatment samples from MM patients (423 ng/ml) was significantly higher than that from the control group (385 ng/ml; *p* = 0.0002) (Figure 5A) or the corresponding samples from LYMPH patients (388 ng/ml; *p* = 0.009). This difference did not seem to be associated with post-HSCT complications. No difference (*p* = 0.56) was found between healthy subjects and patients suffering from lymphoma (Figure 5B).

No significant differences in median CL-LK concentration between males and females were apparent in MM patients (420 vs. 429 ng/ml) or healthy controls (368 vs. 400 ng/ml). However, this value was significantly higher in LYMPH group females (430 vs. 371 ng/ml) compared with males (*p* = 0.004).

The highest CL-LK serum levels (>583 ng/ml) occurred as frequently among MM patients (7%) and LYMPH patients (6%) as controls (5.6%). However, low (< 242 ng/ml) values were under-represented within the MM group (2.1%) compared with the C (5.6%, *p* = 0.002) or LYMPH (6%, *p* = 0.037) groups. It should be stressed that low CL-LK was found in none of the MM patients experiencing infections or febrile neutropenia (Table 7).

**Table 7:** Frequency of low serum concentrations of collectin CL-LK in investigated groups (for MM and LYMPH groups: data from sample 1, taken before chemotherapy).

**Group *(n*)**	**% of concentrations < 242 ng/ml**	**Statistical significance (vs. C)**
C (227)	9.7	-
MM (187)^*^	2.1	*p* = 0.002; OR 0.2; 95% CI (0.07-0.6)
MM-A (41)	0	*p* = 0.031; OR 0.11; 95% CI (0.007-1.85)
MM-B (30)	0	*p* = 0.087
MM-A+B (71)	0	*p* = 0.003; OR 0.06; 95% CI (0.004-1.07)
MM-C (38)	0	*p* = 0.052
MM-D (78)	5.1	*p* = 0.25
LYMPH (116)^*^	7.8	*p* = 0.69
LYMPH-A (32)	9.4	*p* = 1
LYMPH-B (38)	7.9	*p* = 1
LYMPH-A+B (70)	8.6	*p* = 1
LYMPH-C (10)	20	*p* = 0.27
LYMPH-D (36)	2.8	*p* = 0.22

* *MM-A, LYMPH-A: patients who experienced infections with proven bacteremia; MM-B, LYMPH-B: patients who experienced infections with no bacteremia; MM-C, LYMPH-C: patients who experienced febrile neutropenia; MM-D, LYMPH-D: patients who experienced none of afore-mentioned complications during hospital stay*.

CL-LK concentration during treatment varied, but the changes noted were not so striking as for MBL. They were however significant when data from MM patients who suffered from bacteremia or FN were analyzed (Friedman's ANOVA: *p* < 0.000001 and *p* = 0.008, respectively) (Figure 3B). Within LYMPH group, variations were significant for persons affected by bacteremia only (*p* = 0.035). The level of CL-LK was generally lower at point 2 (before HSCT) compared with preceding and following samples. Interestingly, data from minority of patients who were sampled during control examinations suggested slight but significant increase of CL-LK at 45 and 100th days after HSCT (443 ng/ml; *n* = 26, *p* = 0.016 and 457 ng/ml; *n* = 23, *p* = 0.048 compared with data from sample 1). In the case of LYMPH, the median corresponding to samples 100 (384 ng/ml) was similar to that from samples taken before chemotherapy (Figure 4B).

Described variations were not associated with changes of blood cell counts or inflammatory markers (Table 6).

An incidental finding was a lack of detectable CL-LK in a healthy 45-year old woman. A repeat sample 2 months later yielded only a trace (< 40 ng/ml). Sequencing of the *COLEC10* gene did not reveal homozygosity for any known variant allele or “new” mutation when compared to the reference gene sequence (NCBI Reference Sequence: NC_000008.11, accessed from: https://www.ncbi.nlm.nih.gov/gene/10584) (not shown).

### Concentration of MASP-2

**Figure 6:**
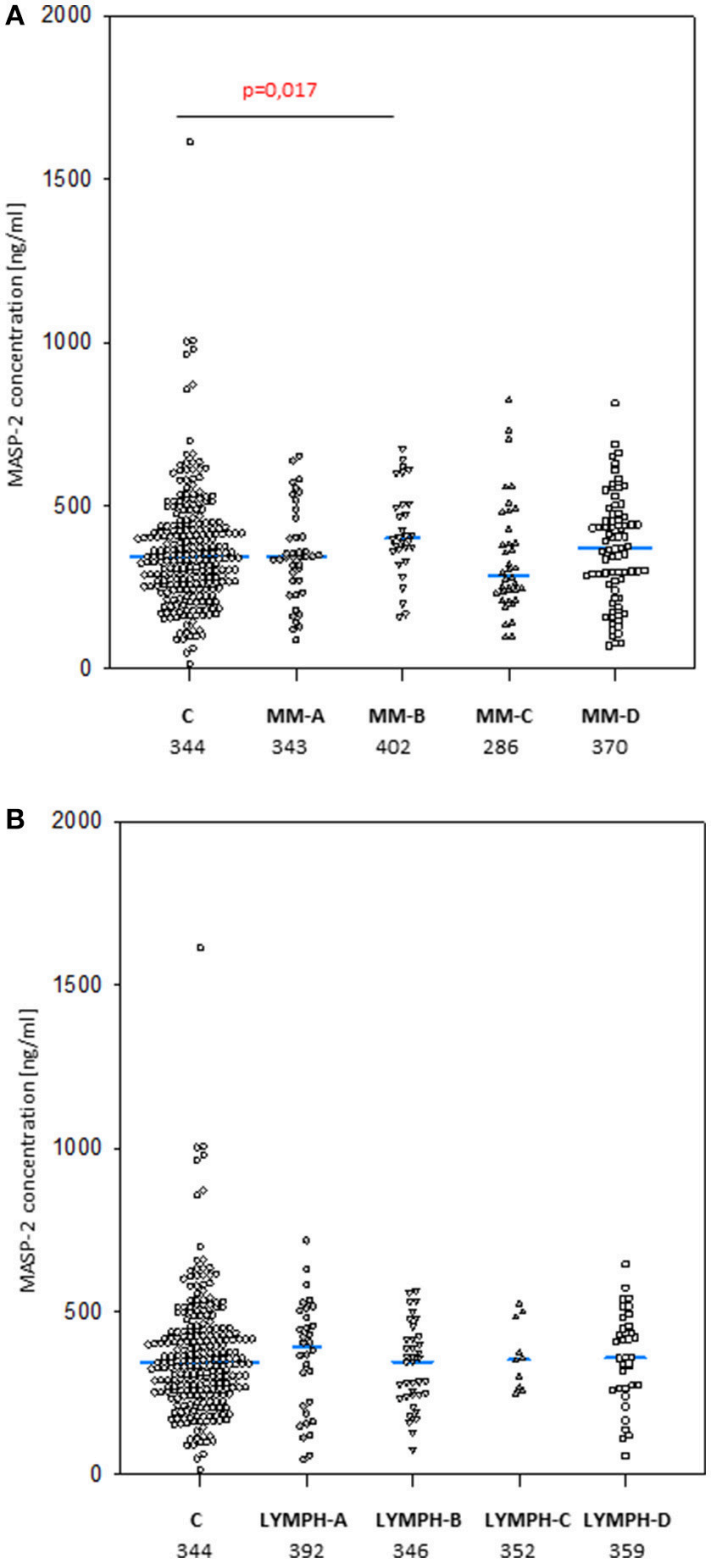
MASP-2 serum concentrations in patients (before conditioning chemotherapy) and controls. Blue bars present median values (given below group descriptions). **(A)** controls (C) vs. multiple myeloma (MM) patients; **(B)**—controls vs. lymphoma (LYMPH) patients. MM-A, LYMPH-A: patients who experienced infections with proven bacteremia; MM-B, LYMPH-B: patients who experienced infections with no bacteremia; MM-C, LYMPH-C: patients who experienced febrile neutropenia; MM-A, LYMPH-A: patients who experienced none of afore-mentioned complications during hospital stay.

No significant differences were found when pre-treatment median MASP-2 serum concentrations from MM (357 ng/ml) or LYMPH (359 ng/ml) were compared with that from healthy controls (344 ng/ml) (Figure 6). However, a higher value was observed in the subgroup of MM patients who had infections with no bacteremia (478 ng/ml, *p* = 0.017 vs. C group). Furthermore, median pre-treatment MASP-2 was higher in MM patients suffering from infections with Gram-positive bacteria (403 ng/ml) relative to those infected by Gram-negative bacteria (343 ng/ml; *p* = 0.008).

Within the control group, a significant sex difference in median MASP-2 level was found (372 vs. 330 ng/ml for males and females, respectively, *p* = 0,019). No such relationship was found in MM (360 vs. 352 ng/ml) or LYMPH (347 vs. 371 ng/ml) groups.

When the frequencies of high (>628 ng/ml) MASP-2 concentrations were compared, no significant difference between the groups was found (C: 5.1%, MM: 6.4%, LYMPH: 2.6%). Similarly, the incidence of low (<172 ng/ml) MASP-2 was similar among healthy subjects (10.2%), MM patients (13.4%), and LYMPH patients (13.8%).

MASP-2 concentrations underwent highly significant treatment-associated changes in both groups of patients, independently of complications (Figure 3C). There was again an increase after treatment, but the kinetics differed from those of MBL. One and three months after discharge, median MASP-2 concentration in LYMPH patients was still higher than at point 1 (386 ng/ml, *n* = 40, *p* = 0.0003 and 419 ng/ml, *n* = 36, *p* = 0.006) while for MM group, the difference was insignificant (Figure 4C).

In both MM and LYMPH groups, MASP-2 correlated positively with CRP and inversely with WBC, ANC and PLT counts (Table 6). In contrast to MBL, serum MASP-2 concentrations did not differ between either group of patients and controls in relation to MASP-2 genotypes (Table 8).

**Table 8:** Serum MASP-2 concentrations in controls and patients (sample 1), corresponding to *MASP2* genotypes (+1111 A>C SNP).

**Genotype**	**Group (*n*)**	**MASP-2 concentrations (ng/ml)**		**Statistical significance (vs. C)**
		**Median**	**IQR**	
A/A	C (171)	312	238–415	–
	MM (120)	320	231–404	*p* = 0.83
	LYMPH (89)	332	233–417	*p* = 0.86
A/C or C/C	C (73)	410	340–513	–
	MM (67)	437	335–544	*p* = 0.46
	LYMPH (27)	455	376–506	*p* = 0.23

## Discussion

Both cancer and its treatment impair immunity, but the role of MBL and the significance of its deficiency in this context are controversial since contrasting and apparently contradictory findings have been reported. Particular attention has focused on the effect of chemotherapy, both during the immediate neutropenic period and during longer periods of follow-up. Here we have demonstrated with a large series of multiple myeloma patients given similar therapy (and more heterogeneous lymphoma group), that MBL deficiency has no influence on the incidence of infections/febrile neutropenia. However, over a 6-month period of follow-up, MBL deficiency was over-represented in the small number of patients experiencing very severe infections. Our findings with a substantially larger series are not inconsistent with the studies of Molle et al. (46, 47) with a broadly similar group of myeloma patients.

These results could be interpreted as meaning MBL has little influence during the period of chemotherapy-induced cytopenia (and does not affect post-HSCT recovery of leukocytes, Supplementary Figure 2), but could have a protective effect when able to act in combination with phagocytic cells. That interpretation could explain why for patients receiving chemotherapy in general, MBL is found to be effective in studies with a medium to long follow-up period (48, 49) but ineffective in short-term follow-up studies (50, 51).

In this study we confirmed the observation of others that chemotherapy is generally followed by an increase in MBL concentration (46, 48, 52). We found that after several weeks it returns to the initial level. A new observation was an association between MBL deficiency and myeloma itself at the genetic level. Furthermore, our data suggest a protective effect of “gt1” from lymphoma. If and when confirmed, this would be the first association of a *MBL2* gene 3′-untranslated region polymorphism with cancer in Europeans [those SNPs were previously reported to have associations with breast or colon cancer in African-Americans but not in Caucasians (53, 54)]. The influence of 3′-end variant on serum MBL concentration was originally reported by Bernig et al. (53, 55). They suggested that Ex4-1067 A haplotype corresponds to high MBL. Indeed, in our study A/A homozygotes (“gt1” genotype) had higher median than A/G heterozygotes (“gt3”) while, consequently, heterozygotes (“gt2”) had higher values than G/G homozygotes (“gt4”) (Figure 2). The Ex4-710/901/1067/1483 SNP were found to be components of a haplotype block (28, 54, 55). Our data (Table 3) are in agreement with that finding. Therefore, in general (although with very few exceptions), analyzing of one polymorphic site may provide information about the others (including also Ex4-718/845/879) (Table 3). Interestingly, the C allele corresponding to Ex4-1483 (associated with Ex4-1067 G) is responsible for interaction with miR-27a, a microRNA molecule which was suspected to predict lower MBL plasma concentration (54, 56). Again, it has been to some extent confirmed here: “gt1” and “gt3” carriers (T/T homozygotes) have generally higher MBL than “gt2” and “gt4” (T/C genotype) (Figure 2). Obviously, an interplay between 3′ block and 5′ one (promoter/5′-UTR/exon 1) has to be taken into account in considering genotype-phenotype relationships (28, 54, 56). For example, strong linkage disequilibria of Ex4-1067 with promoter H/L and Y/X SNP were proven [LDmatrix (https://analysistools.nci.nih.gov) (57)]. It is therefore noteworthy that the majority of “gt1” genotype carriers (74/95 controls and 61 of 87 patients) had YA/YA (“high MBL-producing”) genotypes. Interestingly, the most prevalent 5′ end variant associated with “gt3” was YA/XA (34/47 controls and 52/69 patients; majority of remaining individuals carried at least one LXA allele) (not shown). That altogether confirms apparent links between Ex4-1067 A and promoter (position−221) Y alleles as well as G and X variants, respectively.

MBL, naturally complexed with MASP has been evidenced (employing animal model) to contribute to mobilization of hematopoietic cells from bone marrow to peripheral blood). That complex is able to trigger both complement and coagulation systems activation, cross-talking in mobilization process (58–60). It has been suggested that MBL-deficient patients may be poor HSPC (hematopoietic stem progenitor cells) mobilizers even upon stimulation for transplantation (58, 59). It however has not been confirmed in our study: most of MBL deficient individuals (confirmed by both genotyping and MBL serum level) responded normally (not shown). It may be speculated that MBL deficiency is, at least partially, compensated by other lectin pathway-associated pattern-recognition molecules when MASP function is normal. Furthermore, it seems not to affect markedly recovery of leukocytes (at least when their counts recorded at ~14th day after HSCT, see Supplementary Figure 2).

Another collectin, CL-LK (as mentioned being a complex of CL-10 and CL-11), was found at higher concentrations in myeloma patients compared with controls. One and three months after discharge (samples 45 and 100) its level exceeded an initial one. However, no clinical significance of extremely high or low CL-LK was apparent. This is the first possible association of CL-LK with a hematological malignancy to have been noted. During hospital stay, CL-LK underwent marked changes in myeloma patients affected by bacteremia or febrile neutropenia suggesting involvement of this collectin in the immune response against some potentially life-threatening events.

The results of a retrospective study indicated that higher MASP-2 serum concentrations are associated with a longer event-free survival (EFS) in children with lymphoma (especially HL) (61). We have conducted a prospective study, and collected data from follow up longer than 3–6 months from a relatively low number of patients. Furthermore most of them survived that period and had no relapse so our results cannot easily be compared with the previous report. As with MBL, higher serum level of its associated serine protease-2 before chemotherapy seemed to be associated with hospital infections, at least in MM patients and Gram-positive bacterial agents. Earlier, *MASP2* variant allele (SNP at position +1111) was suggested to be protective against DLBCL (62). Among 31 DLBCL patients studied here, 7 (22.6%) were heterozygotes (no difference in comparison to C group). It should however be stressed that relatively high incidence of *MASP2* +359 A/G heterozygosity was noted among LYMPH patients who experienced bacteremia while relatively low - of +1111 A/C heterozygosity in LYMPH group in general. Both mentioned genotypes influence MASP-2 serum concentration (but not MBL-MASP-2 complex activity when measured as C4 cleaving potency; not shown). The *MASP2* +359 G variant abolishes the formation of the MBL-MASP-2 complex and heterozygotes present approx. half MASP-2 concentration in serum. However, this level suffices for full activity of the MBL pathway (37).

Cancers studied here seem in general to affect concentrations of complement activating collectins and MASP-2 disparately in males and females. As previously demonstrated by Troldborg et al. (63), MBL, CL-10, and CL-11 average levels do not differ between men and women while MASP-2 is higher in men. That was fully confirmed here for controls only. Neither in MM nor LYMPH group the latter differed depending on sex while MBL was found significantly higher in MM males compared with females and opposite effect was noted for CL-LK.

To summarize, we have studied the complex cross-talk between numerous endogenous and exogenous factors in the process of treating patients suffering from MM and LYMPH. Monitoring was started immediately before conditioning chemotherapy and continued for several weeks. The investigated factors, involved in activation of the complement and coagulation cascades, are undoubtedly important players in the events investigated, influencing disease/treatment course and outcome and also being influenced by disease itself and therapeutic procedures. Our data indicate multiple MBL involvements. We found its primary deficiency to be associated with MM risk but not hospital infections during cytopenia. Moreover, high MBL-conferring genotypes and high serum MBL were to some extent associated with adverse effects. Later, during follow-up, MBL deficiency seemed to predict higher morbidity and mortality due to infections. Additionally, we reported the first possible clinical association of a *MBL2* 3′-UTR polymorphism (protective effect of “gt1” from lymphoma) in Caucasians.

## Author contributions

MC, KJ, AW, ASŚ co-authored the project and designed the study. ASŚ and MC planned and supervised experimental work which was done by ASŚ, AS, MM, AS-P, ŁE, and KK. AW, SG, and KJ supervised qualification and recruitment of patients and/or controls. MN, IM, AS-K, MS-K, KM, OB, and AG were responsible for patients' qualification, taking and collecting samples as well as collecting clinical data/follow-up. MLK and AG qualified controls, provided DNA/serum samples and corresponding clinical data. ST and JCJ produced anti-CL-10 and anti-MASP-2 monoclonal antibodies and discussed the data. MC and MM performed the statistical analysis. AS revised the data. MC wrote the original draft of the paper. All authors contributed to the manuscript revision/correction and approved the version to be submitted.

### Conflict of interest statement

The authors declare that the research was conducted in the absence of any commercial or financial relationships that could be construed as a potential conflict of interest.

## Abbreviations

DLBCL: diffuse large B-cell lymphoma
HL: Hodgkin's lymphoma
HSCT: hematopoietic stem cell transplantation
LYMPH: lymphoma
MM: multiple myeloma
NHL: non-Hodgkin's lymphoma.

## References

[1] Hari P. Recent advances in understanding multiple myeloma. Hematol Oncol Stem Cell Ther. (2017) 10:267–71. 10.1016/j.hemonc.2017.05.005 28633036

[2] Stevenson JD Wall C Patel A Lim J Multiple myeloma: a review. Orthop Trauma (2014) 28:187–93. 10.1016/j.mporth.2014.05.007

[3] Rölling C Knap S Bornhäuser M Multiple myeloma. Lancet (2015) 385:2197–208. 10.1016/S0140-6736(14)60493-1 25540889

[4] Kumar SK Rajkumar V Kyle RA van Duin M Sonneveld P Mateos MV Multiple myeloma. Nat Rev Dis Primers (2017) 3:17046:10.1038/nrdp.2017.46. 28726797

[5] Brigle K Rogers B. Pathobiology and diagnosis of multiple myeloma. Semin Oncol Nurs. (2017) 33:225–36. 10.1016/j.soncn.2017.05.012 28688533

[6] Shanbhag S Ambinder AF. Hodgkin lymphoma: a review and update on recent progress. CA Cancer J Clin. (2018) 68:116–32. 10.3322/caac.21438 29194581 PMC5842098

[7] Pileri SA Ascani S Leoncini L Sabattini E Zinzani PL Piccaluga PP. Hodgkin's lymphoma: the pathologist's viewpoint. J Clin Pathol. (2002) 55:162–76. 11896065 10.1136/jcp.55.3.162 PMC1769601

[8] Townsend W Linch D. Hodgkin's lymphoma in adults. Lancet (2012) 380:836–47. 10.1016/S0140-6736(12)60035-X 22835602

[9] Watkins MP Fanale MA Bartlett NL. SOHO state of the art updates and next questions: Hodgkin lymphoma. Clin Lymphoma Myeloma Leuk. (2018) 18:81–90. 10.1016/j.clml.2018.01.001 29366607

[10] Ninkovic S Lambert J Non-Hodgkin lymphoma. Medicine (2017) 45:297–304. 10.1016/j.mpmed.2017.02.008

[11] Warzocha K Lech-Marańda E Diagnosis and treatment of non-Hodgkin lymphomas. Post N Med. (2011) XXIV:567–76. Available online at: http://www.pnmedycznych.pl/wp-content/uploads/2014/09/pnm_2011_567_576.pdf

[12] Shankland KR Armitage JO Hancock BW. Non-Hodgkin lymphoma. Lancet (2012) 380:848–57. 10.1016/S0140-6736(12)60605-9 22835603

[13] Lowry L Linch D Non-Hodgkin's lymphoma. Medicine (2013) 41:282–9. 10.1016/j.mpmed.2013.03.008

[14] Skarbek P Turner D Seftel M Epidemiology of non-Hodgkin lymphoma. Transfus Apher Sci. (2013) 49:133–8. 10.1016/j.transci.2013.07.014 23958141

[15] Armitage JO Gascoine RD Lunning MA Cavalli F. Non-Hodgkin lymphoma. Lancet (2017) 390:298–310. 10.1016/S0140-6736(16)32407-2 28153383

[16] Terpos E on behalf of the International Myeloma Society. Multiple myeloma: clinical updates from the American Society of Hematology Annual Meeting 2016. Clin Lymphoma Myeloma Leuk. (2017) 17:329–39. 10.1016/j.clml.2017.02.010 28462890

[17] Safdar A Armstrong D. Infections in patients with hematologic neoplasms and hematopoietic stem cell transplantation: neutropenia, humoral, and splenic defects. Clin Infect Dis. (2011) 53:798–06. 10.1093/cid/cir492 21890754

[18] Khayr W Haddad RY Noor SA. Infections in hematological malignancies. Dis Mon. (2012) 58:239–49. 10.1016/j.disamonth.2012.01.001 22449371

[19] Balletto E Mikulska M. Bacterial infections in hematopoietic stem cell transplant recipients. Mediterr J Hematol Infect Dis. (2015) 7:e2015045. 10.4084/MJHID.2015.045 26185610 PMC4500472

[20] Ogonek J Kraij Juric M Ghimire S Varanasi PR Holler E Greinix H. Immune reconstitution after allogeneic stem cell transplantation. Front Immunol. (2016) 7:507. 10.3389/fimmu.2016.00507. 27909435 PMC5112259

[21] Pankratova OS Chukhlovin AB Time course of immune recovery and viral reactivation following hematopoietic stem cell transplantation. Cell Therap Transplant. (2016) 5:32–42. 10.18620/ctt-1866-8836-2016-5-4-32-43

[22] Ruhnke M Arnold R Gastmeier P. Infection control issues in patients with haematological malignancies in the era of multidrug-resistant bacteria. Lancet Oncol. (2014) 15:e606–9. 10.1016/S1470-2045(14)70344-4 25456379

[23] Markiewski M Lambris JD. Is complement good or bad for cancer patients? a new perspective on an old dilemma. Trends Immunol. (2009) 30:286–92. 10.1016/j.it.2009.04.002 19428302 PMC2704572

[24] Swierzko AS Kilpatrick DC Cedzynski M. Mannan-binding lectin in malignancy. Mol Immunol. (2012) 55:16–21. 10.1016/j.molimm.2012.09.005 23062612

[25] Kjaer TR Le LTM Pedersen JS Sander B Golas MM Jensenius JC. Structural insights into the initiating complex of the lectin pathway of complement activation. Structure (2015) 23:342–51. 10.1016/j.str.2014.10.024 25579818

[26] Henriksen ML Madsen KL Skjoedt K Hansen S. Calcium-sensitive immunoaffinity chromatography: gentle and highly specific retrieval of a scarce plasma antigen, collectin-LK (CL-LK). J Immunol Methods (2014) 413:25–31. 10.1016/j.jim.2014.07.006 25064149

[27] Hansen SW Ohtani K Roy N Wakamiya N. The collectins CL-L1, CL-K1 and CL-P1 and their roles in complement and innate immunity. Immunobiology (2016) 221:1058–67. 10.1016/j.imbio.2016.05.012 27377710

[28] Bernig T Taylor JG Foster CB Staats B Yeager M Chanock SJ. Sequence analysis of the mannose-binding lectin (*MBL2*) gene reveals a high degree of heterozygosity with evidence of selection. Genes Immun. (2004) 5:461–76. 10.1038/sj.gene.6364116 15306844

[29] Seyfarth J Garred P Madsen HO. The “involution” of mannose-binding lectin. Hum Mol Genet. (2005) 14:2859–69. 10.1093/hmg/ddi318 16115813

[30] Verdu P Barreiro LB Patin E Gessain A Cassar O Kidd JR. Evolutionary insights into the high worldwide prevalence of *MBL2* deficiency alleles. Hum Mol Genet. (2006) 15:2650–8. 10.1093/hmg/ddl193 16885193

[31] Van de Geijn FE Dolhain RJEM van Rijs W Willemsen SP Hazes JMW de Groot CJM Mannose-binding lectin genotypes are associated with shorter gestational age. an evolutionary advantage of low MBL production genotypes? Mol Immunol. (2008) 45:1514–8. 10.1016/j.molimm.2007.08.021 17942155

[32] Tomaiuolo R Ruocco A Salapete C Carru C Baggio G Franceschi C. Activity of mannose-binding lectin in centenarians. Aging Cell (2012) 11:394–400. 10.1111/j.1474-9726.2012.00793.x 22239660 PMC3935210

[33] Scorza M Liguori R Elce A Salvatore F Castaldo G. Biological role of mannose-binding lectin: from newborns to centenarians. Clin Chim Acta (2015) 451(Pt A):78–81. 10.1016/j.cca.2015.03.007 25783214

[34] Peterslund NA Koch C Jensenius JC Thiel S. Association between deficiency of mannose-binding lectin and severe infections after chemotherapy. Lancet (2001) 358:637–8. 10.1016/S0140-6736(01)05785-3 11530153

[35] Bak-Romaniszyn L Szala A Sokolowska A Mierzwa G Czerwionka-Szaflarska M Swierzko AS. Mannan-binding lectin deficiency in pediatric patients with inflammatory bowel disease. Scand J Gastroenterol. (2011) 46:1275–8. 10.3109/00365521.2011.594087 21702710

[36] Weckx S Del-Favero J Rademakers R Claes L Cruts M De Jonghe P. NovoSNP, a novel computational tool for sequence variation discovery. Genome Res. (2005) 15:436–42. 10.1101/gr.2754005 15741513 PMC551570

[37] Swierzko ASt Cedzynski M Domzalska-Popadiuk I MacDonald SL Borkowska-Klos M Mannan-binding lectin-associated serine protease-2 (MASP-2) in a large cohort of neonates and its clinical associations. Mol Immunol. (2009) 46:1696–701. 10.1016/j.molimm.2009.02.022 19307021

[38] Cedzynski M Szemraj J Swierzko AS Bak-Romaniszyn L Banasik M Zeman K. Mannan-binding lectin insufficiency in children with recurrent infections of the respiratory system. Clin Exp Immunol. (2004) 136:304–11. 10.1111/j.1365-2249.2004.02453.x 15086395 PMC1809017

[39] Presanis JS Hajela K Ambrus G Gal P Sim RB. Differential substrate and inhibitor profiles for human MASP-1 and MASP-2. Mol Immunol. (2004) 40:921–9. 10.1016/j.molimm.2003.10.013 14725788

[40] Swierzko ASt Szala A Sawicki S Szemraj J Sniadecki M Sokolowska A Mannose-Binding Lectin (MBL) and MBL-associated serine protease-2 (MASP-2) in women with malignant and benign ovarian tumours. Cancer Immunol Immunother. (2014) 11:1129–40. 10.1007/s00262-014-1579-y PMC4209098 25038892

[41] Petersen SV Thiel S Jensen L Steffensen R Jensenius JC. An assay for the mannan-binding lectin pathway of complement activation. J Immunol Methods (2001) 257:107–16. 10.1016/S0022-1759(01)00453-7 11687244

[42] Swierzko ASt Szala A Cedzynski M Domzalska-Popadiuk I Borkowska-Klos M Jopek A. Mannan-binding lectin genotypes and genotype-phenotype relationships in a large cohort of Polish neonates. Hum Immunol. (2009) 70:68–72. 10.1016/j.humimm.2008.10.004 18957309

[43] Swierzko ASt Atkinson APM Cedzynski M MacDonald SL Szala A Domzalska-Popadiuk I. Two factors of the lectin pathway of complement, L-ficolin and mannan-binding lectin, and their associations with prematurity, low birthweight and infections in a large cohort of Polish neonates. Mol Immunol. (2009) 46:551–8. 10.1016/j.molimm.2008.07.025 18950864

[44] Axelgaard E Jensen L Dyrlund TF Nielsen HJ Enghild JJ Thiel S. Investigations on collectin liver 1. J Biol Chem. (2013) 288:23407–20. 10.1074/jbc.M113.492603 23814060 PMC3743509

[45] Moller-Kristensen M Jensenius JC Jensen L Thielens N Rossi V Arlaud G. Levels of mannan-binding lectin-associated serine protease-2 in healthy individuals. J Immunol Methods (2003) 282:159–67. 10.1016/j.jim.2003.08.012 14604549

[46] Molle I Peterslund NA Thiel S Steffensen R. *MBL2* polymorphism and risk of severe infections in multiple myeloma patients receiving high-dose melphalan and autologous stem cell transplantation. Bone Marrow Transpl. (2006) 38:555–60. 10.1038/sj.bmt.1705466 16953214

[47] Molle I Steffensen R Thiel S Peterslund NA. Chemotherapy-related infections in patients with multiple myeloma: associations with mannan-binding lectin genotypes. Eur J Hematol. (2006) 77:19–26. 10.1111/j.1600-0609.2006.00669.x 16827883

[48] Mullighan CG Heatley SL Danner S Dean MM Doherty K Hahn U Mannose-binding lectin status is associated with risk of major infection following myeloablative sibiling allogeneic hemopoietic stem cell transplantation. Blood (2008) 112:2120–8. 10.1182/blood-2007-07-100222 18552214

[49] Horiuchi T Gondo H Miyagawa H Otsuka J Inaba S Nagafuji K. Association of MBL gene polymorphisms with major bacterial infection in patients treated with high-dose chemotherapy and autologous PBSCT. Genes Immun. (2005) 6:162–6. 10.1038/sj.gene.6364165 15674393

[50] Wong M Ohrmalm L Brollden K Aust C Hibberd M Tolfvenstam T Mannose-binding lectin 2 polymorphisms do not influence frequency and type of infection in adults with chemotherapy induced neutropaenia. PLoS ONE (2012) 7:e30819 10.1371/journal.pone.0030819 22363494 PMC3281882

[51] Bergmann OJ Christiansen M Laursen I Bang P Hansen NE Ellegaard J. Low levels of mannose-binding lectin do not affect occurrence of severe infections or duration of fever in acute myeloid leukaemia during remission induction therapy. Eur J Haematol. (2003) 70:91–7. 10.1034/j.1600-0609.2003.00012.x 12581190

[52] Kilpatrick DC McLintock LA Allan EK Kopland M Fujita T Jordanides NE. No strong relationship between mannan binding lectin or plasma ficolins and chemotherapy-related infections. Clin Exp Immunol. (2003) 134:279–84. 10.1046/j.1365-2249.2003.02284.x 14616788 PMC1808868

[53] Bernig T Boersma BJ Howe TM Welch R Yadavalli S Staats B. The mannose-binding lectin (*MBL2*) haplotype and breast cancer: an association study in African-American and Caucasian women. Carcinogenesis (2007) 28:828–36. 10.1093/carcin/bgl198 17071626

[54] Zanetti KA Haznadar M Welsh JA Robles AI Ryan BM McClary AC 3'UTR and functional secretor haplotypes in mannose-binding lectin 2 are associated with increased colon cancer risk in African Americans. Cancer Res. (2012) 72:1467–77. 10.1158/0008-5472.CAN-11-3073 22282660 PMC3306468

[55] Bernig T Breunis W Brouwer N Hutchinson A Welch R Roos D. An analysis of genetic variation across the *MBL2* locus in Dutch Caucasians indicates that 3' haplotypes could modify circulating levels of mannose-binding lectin. Hum Genet. (2005) 118:404–15. 10.1007/s00439-005-0053-5 16208516

[56] Kalia N Sharma A Kaur M Singh Kamboj S Singh J. A comprehensive in silico analysis of non-synonymous and regulatory SNPs of human *MBL2* gene. SpringerPlus (2016) 5:811. 10.1186/s40064-016-2543-4 27390651 PMC4916122

[57] Machiela MJ Chanock SJ. LDlink a web-based application for exploring population-specific haplotype structure and linking correlated alleles of possible functional variants. Bioinformatics (2015) 31:3555–7. 10.1093/bioinformatics/btv402 26139635 PMC4626747

[58] Adamiak M Abdelbaset-Ismail A Suszynska M Abdel-Latif A Ratajczak J Ratajczak MZ. Novel evidence that the mannan-binding lectin pathway of complement activation plays a pivotal role in triggering mobilization of hematopoietic stem/progenitor cells by activation of both the complement and coagulation cascades. Leukemia (2017) 31:262–65. 10.1038/leu.2016.278 27733776 PMC5214582

[59] Adamiak M Abdel-Latif A Ratajczak MZ Mannan binding lectin triggers mobilization of hematopoietic cells. Oncotarget (2017) 43:73368–9. 10.18632/oncotarget.20705 PMC5650267 29088712

[60] Borkowska S Suszynska M Mierzejewska K Ismail A Budkowska M Salata D. Novel evidence that crosstalk between the complement, coagulation and fibrinolysis proteolytic cascades is involved in mobilization of hematopoietic stem/progenitor cells (HSPCs). Leukemia (2014) 28:2148–54. 10.1038/leu.2014.115 24667943 PMC4177021

[61] Zehnder A Fisch U Hirt A Niggli FK Simon A Ozsahin H. Prognosis in pediatric hematologic malignancies is associated with serum concentration of mannose-binding lectin-associated serine protease-2 (MASP-2). Pediatr Blood Cancer (2009) 53:53–7. 10.1002/pbc.22028 19343776

[62] Hu W Bassig BA Xu J Zheng T Zhang Y Berndt SI. Polymorphisms in pattern-recognition genes in the innate immunity system and risk of non-Hodgkin lymphoma. Environ Mol Mutagen (2013) 54:72–7. 10.1002/em.21739 23055202 PMC6800161

[63] Troldborg A Hansen A Hansen SWK Jensenius JC Stengaard-Pedersen K Thiel S. Lectin complement pathway proteins in healthy individuals. Clin Exp Immunol. (2016) 188:138–47. 10.1111/cei.12909 27925159 PMC5343365

