# Supplementary material for: The Role of Complement Activating Collectins and Associated Serine Proteases in Patients With Hematological Malignancies, Receiving High-Dose Chemotherapy, and Autologous Hematopoietic Stem Cell Transplantations (Auto-HSCT)
Source: Front Immunol. 2018 Sep 20;9:2153. doi: 10.3389/fimmu.2018.02153 (PMC6158352; doi:10.3389/fimmu.2018.02153)
Supplement: Supplementary file 1 [file Data_Sheet_1.docx]

Supplementary Material

**Complement activating collectins and associated serine proteases influence disease course in patients with haematological malignancies, receiving high-dose chemotherapy and autologous haematopoietic stem cell transplantations (auto-HSCT)**

Anna S. Świerzko, Mateusz Michalski, Anna Sokołowska, Mateusz Nowicki, Łukasz Eppa, Agnieszka Szala-Poździej, Iwona Mitrus, Anna Szmigielska-Kapłon, Małgorzata Sobczyk-Kruszelnicka, Katarzyna Michalak, Aleksandra Gołos, Agnieszka Wierzbowska, Sebastian Giebel, Krzysztof Jamroziak, Marek L. Kowalski, Olga Brzezińska, Steffen Thiel, Jens C. Jensenius, Katarzyna Kasperkiewicz, Maciej Cedzyński*

* - correspondence: Maciej Cedzyński: mcedzynski@cbm.pan.pl

# Supplementary Data

Reported in our paper data concerning collectin-10 encouraged us to investigate polymorphisms of the *COLEC11* gene, for another complement-activating molecule, collectin-11. As mentioned in the main text, CL-10 and CL-11 exist in circulation as heterodimers (enamed CL-LK) further complexed with MASPs and therefore it is reasonable to investigate both pattern-recognition molecules.

*COLEC11* gene polymorphisms located within exon 7 were investigated with the use of direct sequencing of PCR products: c.505 T>C (p.Ser169Pro, rs387907075), c.610 G>A (p.Gly204Ser, rs387907076) and c.656 A>G, p.His219Arg, rs7567833. The variant allele for 610 G>A SNP was found in none of patients or controls. One of 243 tested controls (female, aged 50 years) was heterozygote for c.505 T>C polymorphism. Furthermore, 11 heterozygotes (c.656 A>G, p.His219Arg, rs7567833) were found among healthy persons, 11 – among 178 MM and 5 among 109 tested LYMPH patients (minor allele frequency: 0.023, 0.031, 0.023, respectively, no significant differences). There was also no association of mentioned *COLEC11* polymorphisms with incidence of complications during hospital stay


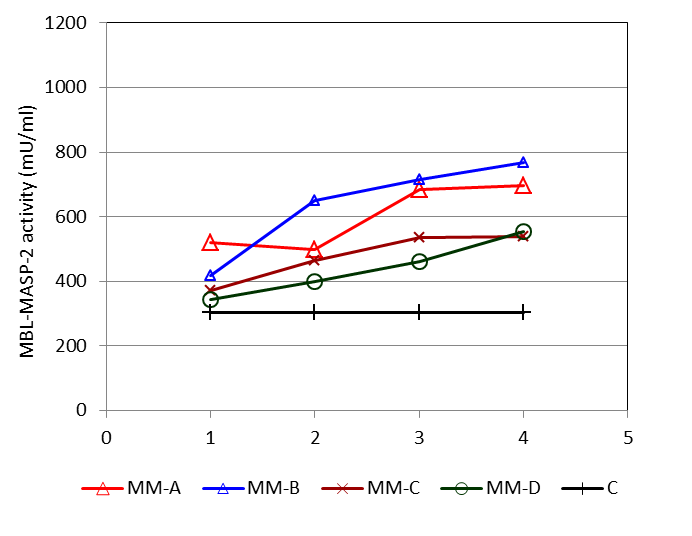

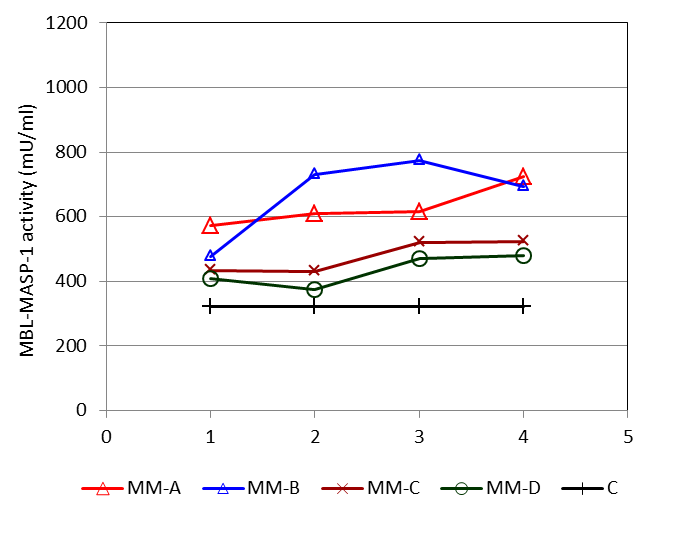


**A**

p=0.0004 MM-A

p=0.00001 MM-C

p<0.000001

p=0.00002 MM-C

p<0.000001 MM-D

* *

* *

* *

* *

* *

p<0.000001 MM-D

p<0.000001 MM-B

*

* *

*

*

* *

* *

*

p<0.000001


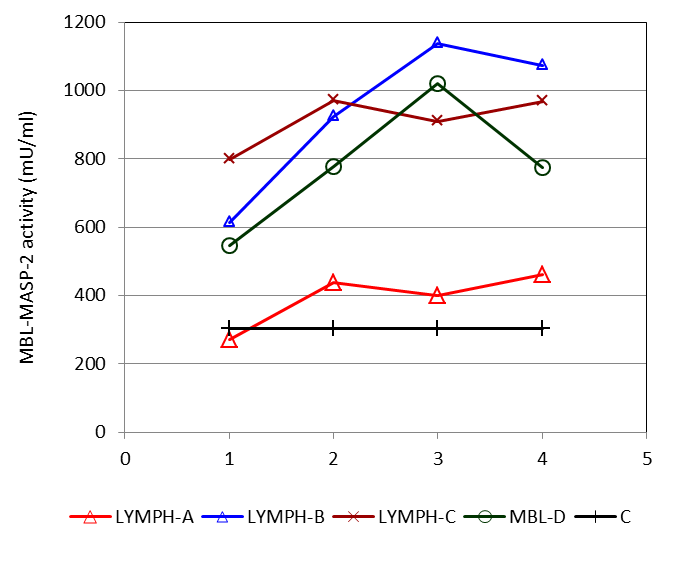

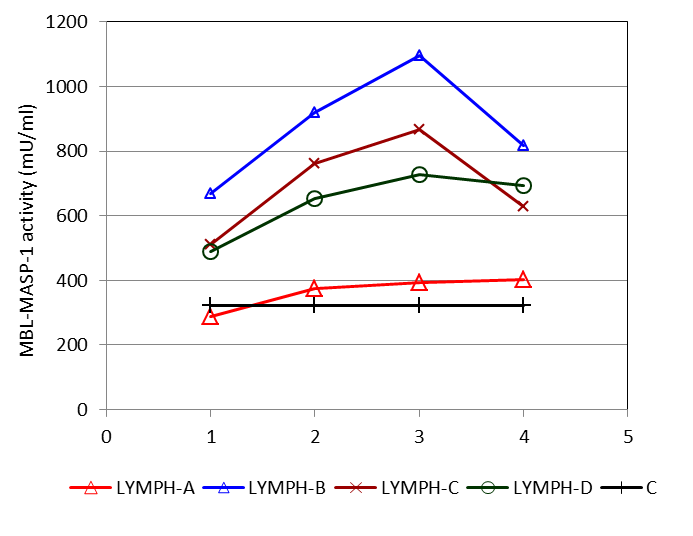


**Supplementary Figure 1.** Changes in activities of MBL-MASP-1 and MBL-MASP-2 complexes in patients suffering from multiple myeloma (**A**) and lymphomas (**B**), during treatment. 1 – blood taken directly before conditioning chemotherapy; 2 – blood taken directly before autologous haematopoietic stem cell transplantation; 3 – blood taken one week after HSCT; 4 – blood taken two weeks after HSCT. Median values for each time-point are presented. MM-A, LYMPH-A: patients who experienced infections with proven bacteremia; MM-B, LYMPH-B: patients who experienced infections with no bacteremia; MM-C, LYMPH-C: patients who experienced febrile neutropenia; MM-D, LYMPH-D: patients who experienced none of afore-mentioned complications during hospital stay. C – controls (sampled once). Statistics: given p values regard to Friedman’s ANOVA while asterisks (in colours corresponding to curves) mark significant differences in comparison with sample 1 (Wilcoxon’s paired sample test).

**B**

p<0.000001

p=0.012 LYMPH-C

p=0.0006 LYMPH-D

p<0.000001 LYMPH-B

*

* * *

* * *

*

*****

*

*

p<0.000001

p=0.018 MM-C

p<0.000001 MM-D

p<0.000001 MM-B

*

*****

*****

*****

* *

* *

* *

ANC x 10^3^/µl

A/A LXA/O + O/O A/A LXA/O + O/O

LYMPH (BEAM) MM (MEL-200)

WBC x 10^3^/µl

A/A LXA/O + O/O A/A LXA/O + O/O

LYMPH (BEAM) MM (MEL-200)

**A**

**B**

PLT x 10^3^/µl

A/A LXA/O + O/O A/A LXA/O + O/O

LYMPH (BEAM) MM (MEL-200)

**Supplementary Figure 2.** Influence of *MBL2* (mannose-binding lectin) genotype on leukocyte count (14 days post-HSCT). **A:** WBC – white blood cells count; **B:** ANC – all neutrophil count; **C:** platelets count. Patients with fully active MBL (A/A genotype) and MBL-deficient (LXA/O or O/O genotype). To avoid effect of different chemotherapy regime and/or radiation; data from patients treated with BEAM (LYMPH) or MEL-200 (MM) were taken only. Blue bars represent median values. No significant differences were noted.

**C**

**Supplementary Table 1.** Aetiological agents of hospital infections in patients.

**A** – infections associated with bacteremia; **B** – infections not associated with bacteremia.

**A**

| Clinical isolates | Number of cases |
| --- | --- |
| *Staphylococcus epidermidis* | 14 |
| *Staphylococcus haemolyticus* | 9 |
| *Staphylococcus hominis* | 9 |
| *Staphylococcus* sp. | 3 |
| Mixed (2 species of staphylococci) | 9 |
| Mixed (staphylococci + other Gram-positive bacteria) | 2 |
| Mixed (staphylococci + Gram-negative bacteria) | 6 |
| Mixed (staphylococci + *Candida* sp.) | 3 |
| *Enterococcus faecalis* | 2 |
| *Enterococcus faecium* | 1 |
| *Micrococcus luteus* | 1 |
| *Clostridium difficile* | 3 |
| *Clostridium sp.* | 1 |
| *Escherichia coli* | 9 |
| Mixed (*E. coli* + enterococci) | 2 |
| *Klebsiella pneumoniae* | 4 |
| Lack of identification | 1 |

**B**

| Clinical isolates | Number of cases |
| --- | --- |
| *Staphylococcus epidermidis* | 1 |
| *Staphylococcus* sp. (coagulase-negative) | 1 |
| Mixed (*Staphylococcus epidermidis* + IAV*) | 1 |
| Mixed (staphylococci + Gram-negative bacteria) | 1 |
| *Enterococcus faecium* | 2 |
| *Clostridium difficile* | 4 |
| *Escherichia coli* | 3 |
| Mixed (*Streptococcus agalactiae + Candida albicans*) | 1 |
| Mixed (*E. coli* + IAV*) | 1 |
| Mixed (*E. coli* + *Klebsiella pneumoniae*) | 3 |
| Mixed (*E. coli* + *Pseudomonas aeruginosa*) | 1 |
| Mixed (*E. coli* + enterococci) | 1 |
| Mixed (*E. coli* + *Candida* sp.) | 1 |
| *Enterobacter* sp. | 1 |
| *Pseudomonas aeruginosa* | 1 |
| *Proteus mirabilis* | 1 |
| *Candida albicans* | 3 |
| *Candida glabrata* | 4 |
| Mixed (2 species of *Candida*) | 3 |
| IAV* | 2 |
| Negative culture/lack of identification | 34 |

* - influenza A virus; diagnosed by RT-PCR
